# Therapeutic efficacy of rhoifolin in type 2 diabetes mellitus: Effects on metabolic parameters, hepatic function, and oxidative stress: a dose-dependent study

**DOI:** 10.3389/fphar.2025.1595323

**Published:** 2025-07-04

**Authors:** Sndos Z. Fattiny, Manal Abdulaziz Binobead, Abu ElGasim A. Yagoub, Ghedeir M. Alshammari, Ali Saleh, Mohammed Abdo Yahya

**Affiliations:** Department of Food Science and Nutrition, College of Food and Agricultural Sciences, King Saud University, Riyadh, Saudi Arabia

**Keywords:** rhoifolin, T2DM, hyperglycemia, hyperlipidemia, inflammation, oxidative stress

## Abstract

**Background/objectives:**

Diabetes is one of the most prevalent chronic disorders globally and is linked to obesity. Research has shown that rhoifolin (ROF) can effectively treat metabolic illnesses. This study examines the impact of ROF on glucose and lipid metabolism in a rat model of Type 2 Diabetes Mellitus (T2DM) and investigates its underlying mechanisms.

**Methods:**

T2DM was induced in adult male Wistar rats by administering a high-fat diet (HFD) along with a low dose of streptozotocin (STZ) (35 mg/kg, i. p.). All experiments were conducted over 8 weeks. Six rat groups (n = 7 per group) were administered either a vehicle or incremental doses of ROF (10, 20, 40 mg/kg) for the last 4 weeks.

**Results:**

ROF significantly improved body weight and protected against hepatic damage and steatosis. It notably reduced plasma glucose, insulin, hemoglobin A1c (HbA1c), and the Homeostasis Model Assessment of Insulin Resistance (HOMA-IR). Serum lipid profiles also improved, with decreases in triglycerides (TGs), cholesterol (CHOL), low-density lipoprotein cholesterol (LDL-c), and free fatty acids (FFAs), and an increase in high-density lipoprotein cholesterol (HDL-c). Hepatic dysfunction was alleviated, as evidenced by normalized levels of aspartate transaminase (AST), alanine transaminase (ALT), and gamma-glutamyl transferase (GGT). ROF reduced inflammation, demonstrated by lower tumor necrosis factor-alpha (TNF-α) and interleukin-6 (IL-6) levels and decreased transcription and nuclear accumulation of nuclear factor kappa beta (NF-κB). It also mitigated oxidative stress, evidenced by reduced malondialdehyde (MDA) and increased glutathione (GSH), superoxide dismutase (SOD), and heme oxygenase-1 (HO-1) levels. ROF normalized hepatic peroxisome proliferator-activated receptor alpha (PPARα) and reduced sterol regulatory element-binding protein 1 (SREBP1) activity. Additionally, it modulated apoptosis by decreasing Bax and caspase-3 while increasing Bcl-2. The treatment of ROF improved hepatic glucokinase (GK) activity and lowered glucose-6-phosphatase (G6Pase) levels. These effects were dose-dependent.

**Conclusion:**

ROF shows significant therapeutic potential by enhancing metabolic parameters and modulating key pathways in T2DM, which can pave the way for future animal and clinical intervention studies to validate its therapeutic efficacy and safety.

## 1 Introduction

High-fat diets (HFDs) are a major driver of the global obesity epidemic, affecting approximately 650 million adults worldwide (World Health Organization, 2020). Excessive caloric intake promotes adipogenesis and disrupts lipid metabolism, leading to obesity and its associated complications. However, obesity is closely linked with insulin resistance (IR), where an impaired cellular response to insulin results in hyperglycemia and serves as a precursor to type 2 diabetes mellitus (T2DM) (Li et al., 2022). As IR progresses, it exacerbates metabolic disturbances, contributing to the development of T2DM and influencing various organ systems beyond glucose regulation (Samuel and Shulman, 2012; Li et al., 2022). Understanding these mechanisms is crucial for developing targeted interventions to mitigate the widespread effects of obesity and T2DM. Indeed, the progression of IR can inflict long-term damage to tissues, particularly the liver, kidneys, and nervous system, through mechanisms that trigger oxidative stress and inflammation (Gregor and Hotamisligil, 2011; Naomi et al., 2023; Hao et al., 2024). These processes underscore the role of hyperglycemia in causing hepatic damage (Brownlee, 2005b; Gregor and Hotamisligil, 2011). Increased inflammatory cytokines from adipose tissue exacerbate hepatic IR, disrupting glucose and lipid metabolism and intensifying hyperglycemia and lipotoxicity (Niranjan et al., 2023). Obese T2DM patients are frequently afflicted by hepatic steatosis and non-alcoholic fatty liver disease (NAFLD) (Godoy-Matos et al., 2020). Elevated levels of circulating free fatty acids initiate an oxidative stress response in hepatocytes by inducing mitochondrial dysfunction and generating reactive oxygen species (ROS) (Masenga et al., 2023; Niranjan et al., 2023). These ROS enhance lipid peroxidation, compromising cellular membranes and organelle function, thereby exacerbating liver injury. Moreover, hyperglycemia contributes to hepatic oxidative stress by activating several ROS-generating pathways, including advanced glycation end-products (AGEs), protein kinase C (PKC), and the polyol pathway (González et al., 2023). Chronic low-grade inflammation, driven by adipose tissue cytokines such as tumor necrosis factor-alpha (TNF-α) and interleukin-6 (IL-6), further aggravates tissue injury by activating pro-inflammatory pathways and recruiting immune cells (Kosmas et al., 2018; Niranjan et al., 2023).

Natural plant-based compounds, including resveratrol, curcumin, berberine, and epigallocatechin gallate, have demonstrated significant potential in treating obesity-related organ damage. These compounds exhibit hypoglycemic and hypolipidemic effects while modulating metabolic pathways, inflammatory cytokines, and antioxidant defenses (Cicero and Baggioni, 2016; Panahi et al., 2021; Wu et al., 2021; Jha et al., 2024). Apigenin, a bioflavonoid found in parsley, celery, and chamomile, shows promising antidiabetic effects through multiple mechanisms. It enhances insulin sensitivity in muscle and adipose tissue, suppresses nuclear factor kappa-beta (NF-κB) and inflammatory cytokines, upregulates anti-oxidants, protects pancreatic β-cells from oxidative stress, and stimulates insulin secretion (Cazarolli et al., 2009; Kang et al., 2011; Suh et al., 2012).

ROF, an apigenin glycoside (apigenin-7-rhamnoglucoside) found in citrus fruits such as grapefruits, lemons, and grapes (Liao et al., 2019), has shown considerable pharmacological efficacy, including anti-oxidant, anti-inflammatory, antiarthritic, and anti-cancer properties. Preclinical studies have highlighted that ROF’s protective effects show substantial pharmacological efficacy, including anti-oxidant, anti-inflammatory, anti-arthritic, and anti-cancer properties in various animal models of toxicity, such as cisplatin-induced damage, arthritis, edema, cardiac dysfunction, epilepsy, and alcoholic liver disease. These effects are primarily attributed to ROF’s antioxidant capacity, its role in reducing ROS, and its ability to suppress inflammatory pathways by targeting NF-κB and reducing cytokine production (El-Shawi and Eldahshan, 2014; Chen et al., 2016; Peng et al., 2020b; Al-Shalabi et al., 2022; Mai et al., 2022b; Zheng et al., 2022; Saher et al., 2023; Akbar et al., 2024). Although the anti-diabetic effects of ROF have been studied less extensively, *in vitro* research indicates its potential. One study demonstrated ROF’s insulin-mimetic action by stimulating adiponectin secretion and Glucose Transporter Type 4 (GLUT-4) expression in adipocytes (Rao et al., 2011a). Another study showed its ability to scavenge free radicals and inhibit amylase activity (Zengin et al., 2023a).

Collectively, these findings suggest that ROF shows potential as a therapeutic agent for managing diabetes mellitus. This study aims to investigate ROF’s hypoglycemic and hypolipidemic effects in a chronic HFD rat model and explore its underlying mechanisms, including its impact on gluconeogenesis, lipogenesis, and hepatic protection through anti-oxidant and anti-inflammatory pathways.

## 2 Materials and methods

### 2.1 Animals

Adult male Wistar rats, all of the same genetic background, aged 6 weeks and weighing 120 ± 10 g, were procured from the animal house of the Animal Facility Department at King Saud University, Riyadh, Saudi Arabia, following ethical approval from the university’s Animal Care and Use Committee (IRB ## KSU-SE-23-34). Rats were housed in groups of seven in standard cages under controlled conditions. The ambient temperature was maintained at 22.5°C ± 1°C with a relative humidity of 55%–60%. A 12-h light-dark cycle was maintained with lights on from 6:00 a.m. to 6:00 p.m. Throughout an initial adaptation period, rats had *ad libitum* access to water and a standard diet containing 13% fats. Environmental enrichment included bedding material, wood shavings, and paper wool nesting to promote natural behaviors. Animal care and experimental procedures adhered to guidelines approved by the university’s animal house veterinarians.

### 2.2 Diets and establishment of T2DM animal model

In our study, we employed HFD and streptozotocin (STZ) to investigate the effects of ROF on metabolic disturbances, liver damage, and the progression of NAFLD in obese rats with T2DM, building on existing literature that demonstrates the utility of this protocol in elucidating metabolic pathways and evaluating potential treatments for obesity/T2DM-related conditions (Zhang et al., 2016; Zhou et al., 2019). This approach ensured a controlled and standardized experimental setup, adhering to ethical guidelines and institutional regulations regarding animal care and use. To induce obesity in our animal model, we utilized the HFD (Cat. #D12451, Research Diets, New Brunswick, NJ, United States), which has been shown to effectively induce metabolic disturbances resembling those observed in human obesity, making it a standard choice in obesity-related research (Panchal et al., 2011). HFD is specifically designed to promote obesity and is characterized by a higher energy density of 4.73 kcal/g (19.8 kJ/g), with 45% of the calories derived from fat, 20% from protein, and 35% from carbohydrates. Herein. The rats were randomly divided and fed the standard diet (STD) or HFD for eight consecutive weeks (the entire study period). At the end of day 27, the HFD rat groups were injected intraperitoneally (i.p.) with STZ (35 mg/kg; dissolved in 0.1 mol/L citrate buffer; pH 4.5), while feeding on HFD until week 8. Three days post-STZ injection, blood was obtained from the rat’s tail after applying local anesthesia. Then, fasting blood glucose (FBG) levels were measured in both groups with a portable glucometer (Açu-check, United States, Roche Diabetes Care). The blood glucose levels of the control rats showed normal fasting glucose levels. However, those HFD rats with FBG levels ≥200 mg/dL were classified as T2DM and subsequently included in our experiments (Noordin et al., 2021; Farrag et al., 2023; Rehman et al., 2023). This protocol has been widely used to induce T2DM in rats (Zhang et al., 2016; Wang et al., 2020; Zhang et al., 2021a; Zhou et al., 2024). The control rats were given a standard diet (Cat. #D12450K, Research Diets, New Brunswick, NJ, United States) for the first 4 weeks, along with injected a single equivalent volume of normal saline as the vehicle on the last day of week 4, and continued on a standard diet until the end of the experiment. This diet is formulated to provide a total energy density of 2.9 kcal/g (12.1 kJ/g), comprising 13% fats, 20% protein, and 67% carbohydrates.

### 2.3 Drugs

ROF powder (99% HPLC) (Code # PHL83302) and carboxymethylcellulose (CMC; Code #C5678) were purchased from Sigma. ROF was freshly dissolved in 0.5% CMC at the desired concentration before use in the experiment.

## 3 Experimental design

**FIGURE 1:**
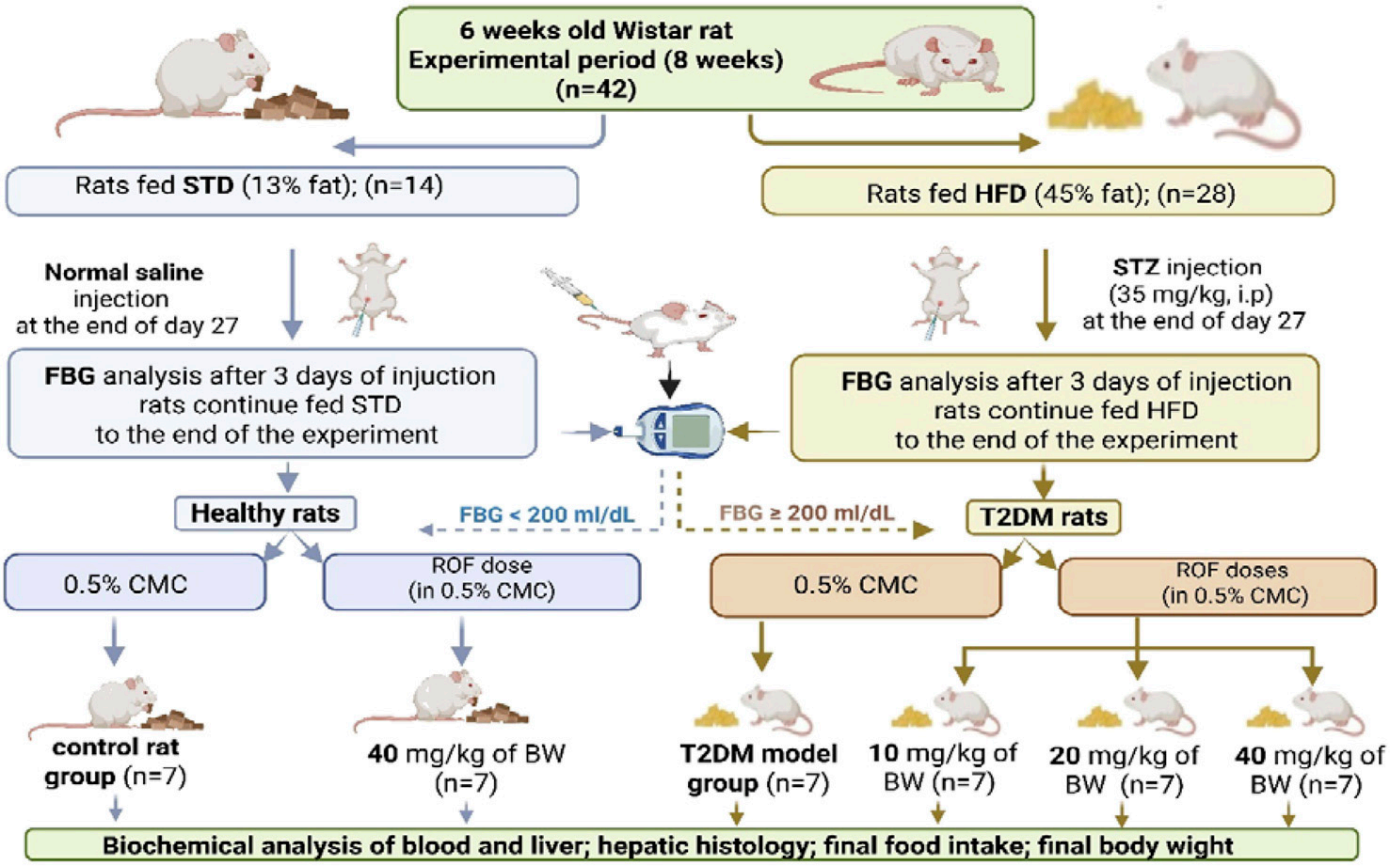
A diagram showing the different experimental groups and experimental design included in this study. Created in BioRender. fattiny, s (2025) https://BioRender.com/92c02c7.

Control rats and those with induced T2DM were randomly assigned to six groups (n = 7 rats/group): (1) The control group: rats were fed a standard diet along with 0.5% CMC (2 mL/day) to serve as a vehicle. (2) The control + ROF-treated group: rats were fed a standard diet and received 40 mg/kg body weight of ROF in 0.5% CMC (2 mL/day). (3) T2DM model rat group: rats with T2DM were fed an HFD and received 0.5% CMC (2 mL/day). (4–6) T2DM + ROF-treated groups: rats with HFD-induced T2DM were orally treated with 10, 20, and 40 mg/kg body weight of ROF in 0.5% CMC (2 mL/day). All experiments were conducted for 8 weeks, and ROF doses were administered daily for the last 4 weeks through oral gavage feeding. A representative diagram of the experimental design and classification of the various groups is shown in Figure 1.

### 3.1 Selection of the doses of ROF

The doses of ROF used in this study were based on the findings from previous studies that examined its efficacy in addressing inflammation and oxidative stress resulting from chronic metabolic diseases. ROF demonstrated a dose-response effect in preventing alcoholic fatty liver in rats, primarily by suppressing inflammatory pathways at doses of 10, 20, and 40 mg/kg (Mai et al., 2022a). Also, increasing doses (10–40 mg/kg) effectively and progressively prevented cardiac damage and dysfunction in mice after exposure to gamma radiation by alleviating lipid peroxidation and oxidative stress, while simultaneously increasing levels of endogenous antioxidants (El-Shawi and Eldahshan, 2014).

### 3.2 Measurement of some anthropometric indicators of obesity

During the final week of the experiment (week 8), rat heights, total body weights, and body mass indices (BMI) were determined. BMI was calculated using the formula [body weight (g) ÷ length^2^ (cm^2^)]. The rat’s body length was measured from the tip of the nose to the anus (in cm) using a non-extensible thread. A pen was used to mark the points between the muzzle and the base of the tail, and the distance between them was measured with a digital caliper or a ruler that had an accuracy of 0.1 cm as described by others (Novelli et al., 2007; Arika et al., 2019; Adebayo et al., 2020).

### 3.3 Euthanasia, blood sampling, and tissue collection

Upon completion of the experimental procedures (on day 60), rats underwent an overnight fasting period before being anesthetized with a combination of ketamine and xylazine (80/10 mg/kg). Blood samples were subsequently collected via cardiac puncture into plain tubes. After allowing a 30-min clotting period at room temperature, the samples underwent centrifugation at 1,200 *g* for 10 min to separate serum, which was then stored at −20 °C for subsequent analysis. Ethical protocols for euthanasia involving neck dislocation were strictly adhered to. Liver tissue and various adipose depots, including subcutaneous (inguinal), epididymal, peritoneal, and mesenteric fat pads, were carefully excised from each rat, weighed, and promptly stored at −80°C until further analysis.

### 3.4 Biochemical measurements in the blood

Serum and plasma samples collected from the study subjects were subjected to comprehensive biochemical analysis utilizing specific ELISA kits validated for rat models. Blood plasma insulin and glucose concentrations were determined using ELISA kits (code # 589501, Ann Arbor, TX, United States, and Code # 10009582, Cayman Chemicals, CA, United States). Serum cholesterol (CHOL) levels were quantified using the ECCH-100 kit from BioAssay Systems, CA, USA. ELISA kits sourced from MyBioSource, CA, United States (code # MBS702165; code # MBS726298 & code #MBS014345) were employed for quantifying free fatty acids (FFAs), low-density lipoprotein-cholesterol (LDL-c), and triglycerides (TG). Serum samples were also analyzed for various biomarkers using specific ELISA kits: tumor necrosis fac-tor-alpha (TNF-α) with code # BMS622 (ThermoFisher, Germany), and interleukin-6 (IL-6) with code #R6000B (R&D Systems, MN, United States). In addition, serum levels of alanine aminotransferase (ALT), gamma-glutamyl transferase (GGT), and aspartate aminotransferase (AST) were evaluated using kits from MyBioSource, San Diego, CA, United States (code # MBS269614 & code # MBS9343646), and Cosmo Bio, CA, United States (code # CSB-E13023r-1), respectively. Duplicate measurements were performed for each sample (n = 7/group) within the experimental groups, adhering rigorously to the manufacturers’ protocols to guarantee precise and reliable data acquisition.

### 3.5 Preparation of liver homogenate

Liver homogenates from frozen rat samples were prepared using a standardized protocol published by Bustin et al. (2009). A homogenization buffer composed of phosphate-buffered saline (PBS) at a 1:10 ratio (w/v) was prepared and supplemented with 1.5 µL of protease and phosphatase inhibitor cocktail (code #ab271306, Abcam, Cambridge, UK) to preserve protein integrity. The liver tissue (100 mg) was cut into small fragments and placed in a pre-cooled homogenizer with the buffer (500 µL). Homogenization was carried out on ice until a uniform consistency was achieved. Subsequently, the homogenate was centrifuged at 1,200 *g* for 10–15 min at 4 °C to remove cellular debris and nuclei. The resulting supernatant was carefully collected, aliquoted into pre-chilled microcentrifuge tubes, and stored at −80 °C until further analysis.

### 3.6 Biochemical analysis in the liver homogenates

ELISA kits were utilized to quantify various biomarkers in the liver. Tumor necrosis factor-alpha (TNF-α) levels were assessed using the kit from ThermoFisher (UK) (code # BMS622), while interleukin-6 (IL-6) levels were measured with the kit from R&D Systems (MN, United States) (code # E-EL-R6000B). Malondialdehyde (MDA), total glutathione (GSH), heme oxygenase-1 (HO-1), and superoxide dismutase (SOD) were quantified using kits (code # EK720188, code # EK720816, code # EK720658, and code # EK720889, respectively) from AFG Scientific (IL, United States). Specific to rats, glucose-6-phosphatase (6-Pase) and glucokinase levels were determined using kits, code # MBS097902 and code # MBS453149, respectively, supplied by MyBioSource (CA, United States). The apoptosis markers Bax, Bcl2, and caspase-3 were measured using the following kits: code #E4513 (BioVision, CA, United States), code # LS-F11016 (LS Bio, MA, United States), and code # LS-F4135 (LS Bio, MA, United States), respectively. Each assay adhered strictly to the manufacturer’s protocols, and measurements were performed in duplicate for n = 7 rats/group.

### 3.7 Preparation of total nuclear cell extract and biochemical analysis

The nuclear fraction from frozen liver samples was isolated using a commercial kit (code # 40010, Active Motif, Tokyo, Japan). In brief, liver tissue was suspended in ice-cold PBS supplemented with a phosphatase inhibitor to minimize protein modifications. The liver tissue was then placed in a hypotonic buffer with 0.05% NP40 detergent and vortexed to lyse the cells and release cytoplasmic proteins. Next, the cytoplasmic fraction was separated, and the nuclei were lysed to solubilize nuclear proteins using a detergent-free lysis buffer supplemented with a protease inhibitor cocktail. In the nuclear fraction, the transcriptional activities of SREBP1 and PPARα, along with the levels of NF-κB, were quantified using rat-specific assay kits (code #ab133125 and code #ab133101, code # ab133112; Abcam, UK, respectively). All assays strictly followed manufacturer protocols and were conducted in duplicate using samples from n = 7 rats per group.

### 3.8 Extraction of lipids from livers and analysis of hepatic lipid levels

The protocol for extracting lipids from the liver followed the classic method developed by Bligh and Dyer (1959). Briefly, a precise weight of frozen liver tissue (100 mg) was homogenized with 1 mL of ice-cold chloroform/methanol (2:1, v/v) in a pre-cold glass tube. After thorough homogenization, the mixture was transferred to a clean glass test tube, and 200 µL of ice-cold water was added and vigorously vortexed for 1 min to induce phase separation. Two phases formed after centrifugation (2000 rpm; 10 min; 4 °C). The lower organic phase, containing the extracted lipids, was carefully transferred to a clean glass test tube. The organic solvent was evaporated under a gentle stream of nitrogen gas, and the resulting dried lipid extract was resuspended in 100 µL of chloroform. All samples were stored at −20 °C until further analysis. The hepatic lipid was analyzed for TGs, CHOL, and FFAs using the same kits used for their measurement in the serum.

### 3.9 Real-time polymerase chain reaction (qPCR)

Quantitative real-time PCR (qPCR) was used to analyze the mRNA levels of lipid-related genes in rat liver tissue. Specific primers targeting NF-κB (XM_342346.4) (For-ward: 5′ GTG​CAG​AAA​GAA​GAC​ATT​GAG​GTG 3′, Reverse: 5′ TCCCGTAACCGCGTA 3′); PPARα (NM_013196.1) (Forward: 5′ TGC​GGA​CTA​CCA​GTA​CTT​AGG​G 3′, Reverse: 5′ GCT​GGA​GAG​AGG​GTG​TCT​GT 3′); and SREBP-1c (NM_001276707.1) (Forward: 5′ GCA​AGG​CCA​TCG​ACT​ACA​TC 3′, Reverse: 5′ TTT​CAT​GCC​CTC​CAT​AGA​CAC 3′) were utilized, as previously described and validated for their specificity. Total RNA extraction from frozen liver samples was performed using the Qiagen RNeasy Mini Kit (Cat. No. 74004, Qiagen, Hilden, Germany), followed by cDNA synthesis with the Thermo Fisher cDNA Synthesis Kit (Cat. No. K1621). RNA quality and concentration were assessed using a Nanodrop spectrophotometer (absorbance ratio at 260/280 nm). qPCR amplification was performed in a Bio-Rad qPCR system using the Ssofast EvaGreen Supermix kit (Cat. No. 172-5200, Bio-Rad, United States). Each 20 μL reaction mixture contained 10 μL of Ssofast EvaGreen master mix, 0.2 µL of both forward and reverse primer (final concentration: 500 nM each), 2 μL of template cDNA (final concentration: 50 ng), and 7.6 µL of nuclease-free water. Amplification conditions involved initial denaturation at 98°C for 30 s, followed by 40 cycles of denaturation at 98°C for 5 s and annealing at 60°C for 5 s. Melting curve analysis was performed to confirm the specificity of amplification. Two samples without template cDNA were included as negative controls. Relative quantification of mRNA expression for each target was analyzed in the machine software using the 2−ΔΔ Ct method (Bustin et al., 2009; Livak and Schmittgen, 2001). Ct values were normalized using β-actin to compare gene expression and transcription levels among different groups. Data analysis was conducted using appropriate software following the manufacturer’s instructions. All experimental procedures strictly adhered to the protocols provided by each kit manufacturer.

### 3.10 Histological study

Hematoxylin and eosin (H&E) staining is a widely used histological technique for visualizing tissue morphology, including liver samples. First, liver tissues fixed in formalin were dehydrated using a series of gradient alcohol concentrations and then embedded in paraffin wax blocks. Liver sections measuring 3–5 μm in thickness were prepared with a microtome and mounted on glass slides. The sections were deparaffinized in xylene and subsequently rehydrated through a series of decreasing ethanol concentrations. Next, the slides were immersed in hematoxylin solution for 5–10 min to stain the nuclei. The slides were then immersed in HCl-alcohol to remove excess stain, followed by a brief rinse with tap water. After that, an eosin solution was applied to stain the cytoplasm and extracellular matrix for 1–3 min. The slides were dehydrated in increasing concentrations of alcohol, cleared with xylene, and mounted with a coverslip using a mounting medium. They were then examined under a light microscope. This procedure ensures optimal staining quality and maintains the tissue structure for histopathological analysis. The protocol follows well-established guidelines in histological research (Bancroft and Gamble, 2008).

### 3.11 Statistical analysis

Data were expressed as means ± standard deviation (SD). Statistical analysis was performed using GraphPad Prism software (version 8, United States). Variations among research groups were evaluated with a one-way ANOVA. Data were significantly differentiated using Tukey’s test at significance levels of p ≤ 0.05.

## 4 Results

### 4.1 Effect of ROF on food intake, body weight, and BMI

The data on food intake, body weight, and BMI across different experimental groups are summarized in Table 1. Control rats exhibited significantly higher final body weight, total fat, and BMI compared to T2DM rats. On the other hand, food intake was lower in the diabetic group than in the control group (p < 0.05).

**TABLE 1:** Variations in body fat and diabetes indicators among the different rat groups.

Parameter	Control	Control + ROF (40 mg/kg)	T2DM	T2DM + ROF (10 mg/kg)	T2DM + ROF (20 mg/kg)	T2DM + ROF (40 mg/kg)
Final body weight (g)	415.1 ± 33.0	442.4 ± 40.0	332.1 ± 24.0^ab^	364.5 ± 32.0^abc^	395 ± 54.20^abcd^	420.0 ± 47.0^cde^
Food intake (last week/g/rat)	265.1 ± 19.8	253.9 ± 22.1	191.1 ± 17.9^ab^	194.5 ± 18.4^ab^	200.3 ± 17.50^ab^	189.3 ± 17.60^ab^
Total fat weight (g)	16.5 ± 1.20	15.9 ± 1.0	9.4 ± 1.10^ab^	11.5 ± 1.10^abc^	13.1 ± 1.20^abcd^	15.6 ± 1.30^cde^
BMI	0.64 ± 0.08	0.61 ± 0.06	0.41 ± 0.03^ab^	0.49 ± 0.04^abc^	0.54 ± 0.05^abcd^	0.65 ± 0.07^cde^
Plasma glucose (mg/dL)	104.4 ± 9.50	110.3 ± 10.4	203.4 ± 18.60^ab^	176.4 ± 15.60^abc^	142.5 ± 12.60^abcd^	106.3 ± 14.0^cde^
Plasma insulin (μU/mL)	4.1 ± 0.87	3.9 ± 0.54	8.1 ± 0.73^ab^	6.3 ± 0.74^abc^	5.2 ± 0.52^abcd^	4.3 ± 0.55^cde^
HOMA-IR	1.05 ± 0.02	1.07 ± 0.01	4.07 ± 0.40^ab^	2.73 ± 0.20^abc^	1.83 ± 0.02^abcd^	1.09 ± 0.01^cde^
Plasma HbA1c (%)	4.2 ± 0.53	4.7 ± 0.61	10.3 ± 1.10^ab^	8.1 ± 0.83^abc^	6.1 ± 0.68^abcd^	4.6 ± 0.51^cde^

Data were analyzed by one-way ANOVA, and means were separated using the Tukey test at different significant levels (p < 0.05). Results are presented as means ± SD, of seven samples per group. Comparison designations are as follows: (a) are denoted as: (a) compared to control, (b) compared to control treated with rhoifolin (40 mg/kg), (c) compared to T2DM, (d) compared to T2DM + rhoifolin (10 mg/kg), and (e) compared to T2DM + rhoifolin (20 mg/kg).

ROF treatment improved body weight in T2DM rats in a dose-dependent manner, with the highest dose (40 mg/kg) resulting in weights comparable to those of control rats. Food intake did not differ significantly with ROF treatment. ROF also increased total fat weight in T2DM rats, with the highest dose approaching levels seen in control rats. ROF treatment improved BMI in T2DM rats. No significant differences were observed between control and control + ROF (40 mg/kg) rats.

### 4.2 Effect of ROF on plasma levels of glucose, insulin, and HbA1c

Table 1 shows plasma glucose, insulin, HbA1c, and HOMA-IR levels across all experimental groups. The rats with T2DM had significantly elevated levels of fasting plasma glucose, insulin, HbA1c, and HOMA-IR compared to the control rats (p < 0.05).

Moreover, administering different doses of ROF (10, 20, and 40 mg/kg) significantly reduced the plasma glucose and insulin levels in T2DM rats. This reduction was dose-dependent, with the 40 mg/kg dose lowering their levels to align with those in the control rats treated with ROF (p < 0.05). The levels of HbA1c and HOMA-IR in rats with T2DM changed similarly when different doses of ROF were given, just as other biomarkers did. The 40 mg/kg dosage in the diabetic group led to HbA1c and HOMA-IR levels comparable to those in the control + ROF group. Conversely, the control + ROF (40 mg/kg) group showed no statistically significant difference in glucose, insulin, HbA1c, and HOMA-IR levels compared to the biomarker levels in the control group. These results indicate the efficacy of ROF on glucose and insulin levels as a key factor in managing and controlling diabetes.

### 4.3 Effect of ROF on serum lipid profile

Table 2 displays the effects of rhoifolin on serum and hepatic lipid profiles as well as liver weights. The results showed that liver weight in the T2DM rat group was higher than in all other groups (p < 0.05).

**TABLE 2:** Analysis of liver weight and lipid metrics in the serum and liver of experimental rat groups.

Sample	Parameter	Control	Control + ROF (40 mg/kg)	T2DM	T2DM + ROF (10 mg/kg)	T2DM + ROF (20 mg/kg)	T2DM + ROF (40 mg/kg)
	Liver weight (g)*	14.6 ± 1.4	13.8 ± 1.5	20.1 ± 1.7^ab^	18.1 ± 1.2^abc^	16.2 ± 1.1^abcd^	14.1 ± 1.2^cde^
Serum	TGs (mg/dL)	83.4 ± 8.1	65.4 ± 8.8^a^	182 ± 16.5^ab^	143.2 ± 13.7^abc^	118.4 ± 10.9^abcd^	89.3 ± 8.4^bcde^
	CHOL (mg/dL)	144.2 ± 12.1	104.5 ± 10.4^a^	278.4 ± 22.5^ab^	231.3 ± 22.3^abc^	202.1 ± 20.9^bcd^	156.3 ± 17.8^bcde^
	LDL-c (mg/dL)	67.4 ± 5.3	54.4 ± 6.1^a^	125.6 ± 14.4^ab^	102.3 ± 11.1^abc^	85.5 ± 7.1^abcd^	64.2 ± 6.1^bcde^
	HDL-c (mg/dL)	41.6 ± 5.1	56.8 ± 6.4^a^	21.2 ± 2.7^ab^	28.5.2 ± 3.4^abc^	35.6 ± 3.7^abcd^	43.2 ± 4.3^bcde^
	FFAs (μmol/L)	338.6 ± 42.1	343.1 ± 35.4	676.2 ± 56.1^ab^	542.2 ± 52.1^abc^	465 ± 43.8^abcd^	371.4 ± 44.3^cde^
Liver	TG (mg/g)	6.5 ± 0.67	4.5 ± 0.58^a^	13.4 ± 1.5 ^ab^	10.2 ± 1.2^abc^	8.5 ± 0.92^abcd^	6.6 ± 0.71^bcde^
	CHOL (mg/g)	4.8 ± 0.41	3.2 ± 0.47^a^	10.4 ± 1.4^ab^	8.4 ± 0.81^abc^	6.13 ± 0.58^abcd^	4.52 ± 0.49^bcde^
	FFAs (μmol/L)	677 ± 61.3	692 ± 59.0	1760 ± 160.0^ab^	1,192 ± 127.0^abc^	8092 ± 90.0^abcd^	639 ± 55.0^cde^

Data were analyzed by one-way ANOVA, and means were separated using the Tukey test at different significant levels (p < 0.05). Results are presented as means ± SD, of seven samples per group. Comparison designations are as follows: (a) compared to the control group, (b) compared to the control group treated with rhoifolin (40 mg/kg), (c) compared to the T2DM, group, (d) compared to the T2DM, rats treated with rhoifolin (10 mg/kg), and (e) compared to the T2DM, rats treated with rhoifolin (20 mg/kg). The measured parameters include triglycerides (TGs), cholesterol (CHOL), low-density lipoprotein cholesterol (LDL-c), free fatty acids (FFAs), and high-density lipoprotein cholesterol (HDL-c).

In the treated diabetic rat groups, the recovery of liver weight was inversely related to varying doses of ROF. Meanwhile, the liver weight of diabetic rats treated with 40 mg/kg ROF was similar to that of the control rats (p > 0.05). There were no significant differences in liver weights between the control group and the control + ROF (40 mg/kg) group.

In contrast, T2DM rats showed increased serum TG, CHOL, and LDL-c levels, along with reduced HDL-c levels compared to the control rats. In T2DM rats, ROF treatment significantly reduced serum TGs, CHOL, FAAs, and LDL-c levels, while increasing HDL-c levels; its effect intensified as the dose concentration increased (p < 0.05). Compared to the control group, the control +40 mg/kg ROF group had significantly lower levels of serum TGs, CHOL, and LDL-c, along with higher levels of HDL-c (p < 0.05), while FFA levels remained unchanged.

Similarly, the hepatic levels of TGs, CHOL, and FAAs were higher in rats with T2DM than those in the control group (p < 0.05). Compared to T2DM rats, diabetic rats treated with different doses of ROF (10, 20, and 40 mg/kg body weight) demonstrated a significant decrease in liver TGs, CHOL, and FAAs (p < 0.05). The levels of these biomarkers in diabetic rats treated with 40 mg/kg of ROF were similar to those in the control rat group. Moreover, compared to the control group, the control +40 mg/kg ROF group showed significantly lower levels of hepatic TGs and CHOL (p < 0.05), while FFA levels remained unchanged. These findings demonstrate that ROF has hypolipidemic features with notable dose-dependent efficacy.

### 4.4 Effect of ROF on serum enzymes of liver function and inflammatory markers

The results of liver function enzymes (AST, ALT, and GTT) and inflammatory markers (TNF-α and IL-6) in the serum are shown in Table 3. The results showed that AST, ALT, and GTT levels were significantly higher in the diabetic group compared to all other groups (p < 0.05).

**TABLE 3:** Hepatic enzyme activities, inflammatory biomarkers, and key glucose metabolism enzymes in experimental rat groups.

Sample	Parameter	Control	Control + ROF (40 mg/kg)	T2DM	T2DM + ROF (10 mg/kg)	T2DM + ROF (20 mg/kg)	T2DM + ROF (40 mg/kg)
	AST (U/l)	31.1 ± 3.7	29.2 ± 12.4	73.4 ± 7.4^ab^	54.3 ± 6.1^abc^	43.6 ± 5.1^abcd^	30.2 ± 3.1^cde^
Serum	ALT (U/l)	55.3 ± 4.3	56.7 ± 6.1	114.3 ± 10.9^ab^	91.3 ± 8.9^abc^	76.5 ± 6.4^abcd^	52.9 ± 5.0^bcde^
	GTT (U/l)	23.2 ± 2.9	25.5 ± 3.2	103.2 ± 10.5^b^	84.5 ± 7.6^abc^	44.3 ± 5.1^bcd^	28.4 ± 3.3^acde^
	TNF-α (pg/mL)	27.4 ± 3.4	25.1 ± 2.5	86.7 ± 8.4^ab^	66.5 ± 5.8^abc^	41.4 ± 5.1^abcd^	27.8 ± 3.3^bcde^
	IL-6 (pg/mL)	47.8 ± 5.5	44.5 ± 5.4	113.2 ± 12.1^ab^	88.9 ± 7.8^abc^	65.6 ± 7.7^abcd^	47.6 ± 5.6^cde^
Liver	GK (pg/mg tissue)	54.3 ± 6.5	51.3 ± 8.5	19.5 ± 1.6^ab^	29.4 ± 2.3^abc^	41.1 ± 4.9^abcd^	56.4 ± 6.8^cde^
	G6Pase (U/mg tissue)	11.8 ± 1.8	10.8 ± 1.3	48.7 ± 3.9^ab^	35.6 ± 3.8^abc^	24.5 ± 2.1^abcd^	11.2 ± 1.6^cde^

Data were analyzed by one-way ANOVA, and means were separated using the Tukey test at different significant levels (p < 0.05). Results are presented as means ± SD, of seven samples per group. Statistical comparisons are denoted as follows: (a) compared to the control, (b) compared to the control treated with rhoifolin (40 mg/kg), (c) compared to T2DM, (d) compared to T2DM, treated with rhoifolin (10 mg/kg), and (e) compared to T2DM, treated with rhoifolin (20 mg/kg). The measured parameters include aspartate transaminase (AST), alanine transaminase (ALT), gamma-glutamyl transferase (GGT), tumor necrosis factor-alpha (TNF-α), interleukin-6 (IL-6), free fatty acids (FFAs), glucokinase (GK), and glucose-6-phosphatase (G6Pase).

ROF treatment significantly decreased elevated levels of AST, ALT, and GTT in rats with T2DM, with improvements correlating to higher dosages (p < 0.05). The enzyme levels in T2DM rats approached normal at the maximum ROF dosage of 40 mg/kg (p < 0.05). On the other hand, treating control rats with ROF (40 mg/kg) did not significantly impact AST, ALT, and GTT levels when compared to the control group.

Regarding proinflammatory cytokines, serum TNF-α and IL-6 levels in T2DM rats were significantly higher than in all other rat groups. After ROF treatment, the levels of these biomarkers for the T2DM rat group significantly decreased in a dose-dependent manner, with the 40 mg/kg dose substantially reducing the biomarker levels to align with those of the control rat groups (p < 0.05). The serum TNF-α and IL-6 levels in the control +40 mg/kg ROF group were similar to those of the control group (Table 3). These results suggest that ROF can mitigate inflammatory and metabolic dysregulations associated with T2DM.

### 4.5 Effect of ROF on liver key enzymes of glucose metabolism

The results for key hepatic glycolytic and gluconeogenesis enzymes (GK and G6Pase, respectively) across all rat groups are presented in Table 3. T2DM rats had significantly lower levels of GK and higher levels of G6Pase compared to the control rats (p < 0.05).

As the ROF dose increased from 10 to 40 mg/kg of body weight, GK levels in T2DM rats gradually increased, with a significant magnitude. The 40 mg/kg ROF-treated T2DM rats had enzyme levels comparable to those in the control rats. In contrast, G6Pase levels in T2DM rats significantly and gradually decreased with increasing ROF doses. Furthermore, the 40 mg/kg dose of ROF resulted in a significant decrease in G6Pase levels in T2DM rats, approaching those of the control rats (p < 0.05). The control +40 mg/kg ROF group did not show significant differences in these enzyme levels compared to the control group. These findings highlighted the effectiveness of ROF in restoring the normal regulation of hepatic enzymes as the dosage increases.

### 4.6 Effect of ROF on hepatic markers of inflammation

**FIGURE 2:**
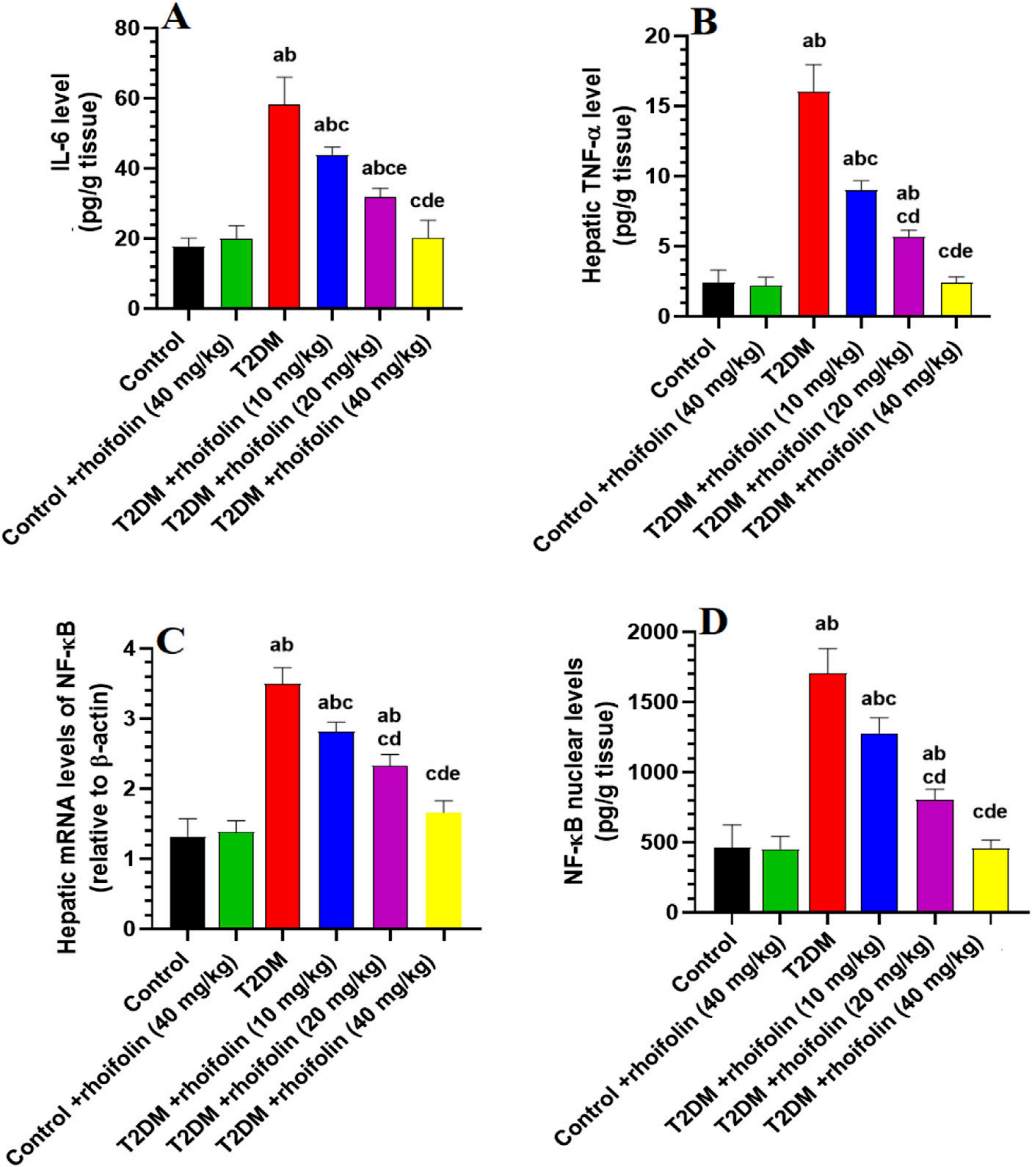
Hepatic profiles of inflammatory biomarkers and transcription factors in experimental rat groups. **(A,B)** represent the hepatic levels of interleukin-6 (IL-6) and tumor necrosis factor-alpha (TNF-α), respectively. **(C,D)** represent the hepatic mRNA and nuclear content of nuclear factor kappa B (NF-κB). Data were analyzed by one-way ANOVA, and means were separated using the Tukey test at different significant levels (p < 0.05). Results are presented as means ± SD of seven samples per group. Statistical contrasts are denoted as: **(a)** against the control group, **(b)** *versus* the control treated with rhoifolin (40 mg/kg), **(c)** compared to the T2DM group, **(d)** compared to T2DM rats treated with rhoifolin at 10 mg/kg, and **(e)** compared to the T2DM group administered rhoifolin at 20 mg/kg.

Figures 2A–D displays the results of pro-inflammatory cytokines TNF-α and IL-6, and mRNA and nuclear NF-κB in the liver. Rats with T2DM had significantly higher levels of hepatic TNF-α and IL-6 than the control rats (p < 0.05). ROF treatment significantly reduced hepatic IL-6 and TNF-α levels in rats with T2DM (p < 0.05). The effect was more significant with the 40 mg/kg dose, resulting in biomarker levels similar to those of the control group. The control +40 mg/kg ROF group and the control group displayed similar IL-6 and TNF-α levels (p > 0.05).

The mRNA NF-ĸB levels were significantly higher in T2DM rats than in the control group. ROF treatment markedly reduced (p < 0.05) the mRNA NF-ĸB levels in the livers of T2DM rats compared to those in the control rats (Figure 2C). Nuclear NF-ĸB levels were significantly increased (p < 0.05) in T2DM rats compared to the control rats. ROF-treated T2DM rats showed a significant reduction in NF-ĸB levels compared to T2DM rats. However, treating the control rats with 40 mg/kg of ROF did not significantly impact the mRNA and nuclear NF-ĸB levels compared to the control (Figure 2D). These results indicate that ROF has anti-inflammatory properties, particularly at 40 mg/kg of body weight (p < 0.05).

### 4.7 Effect of ROF on hepatic markers of oxidative stress

**FIGURE 3:**
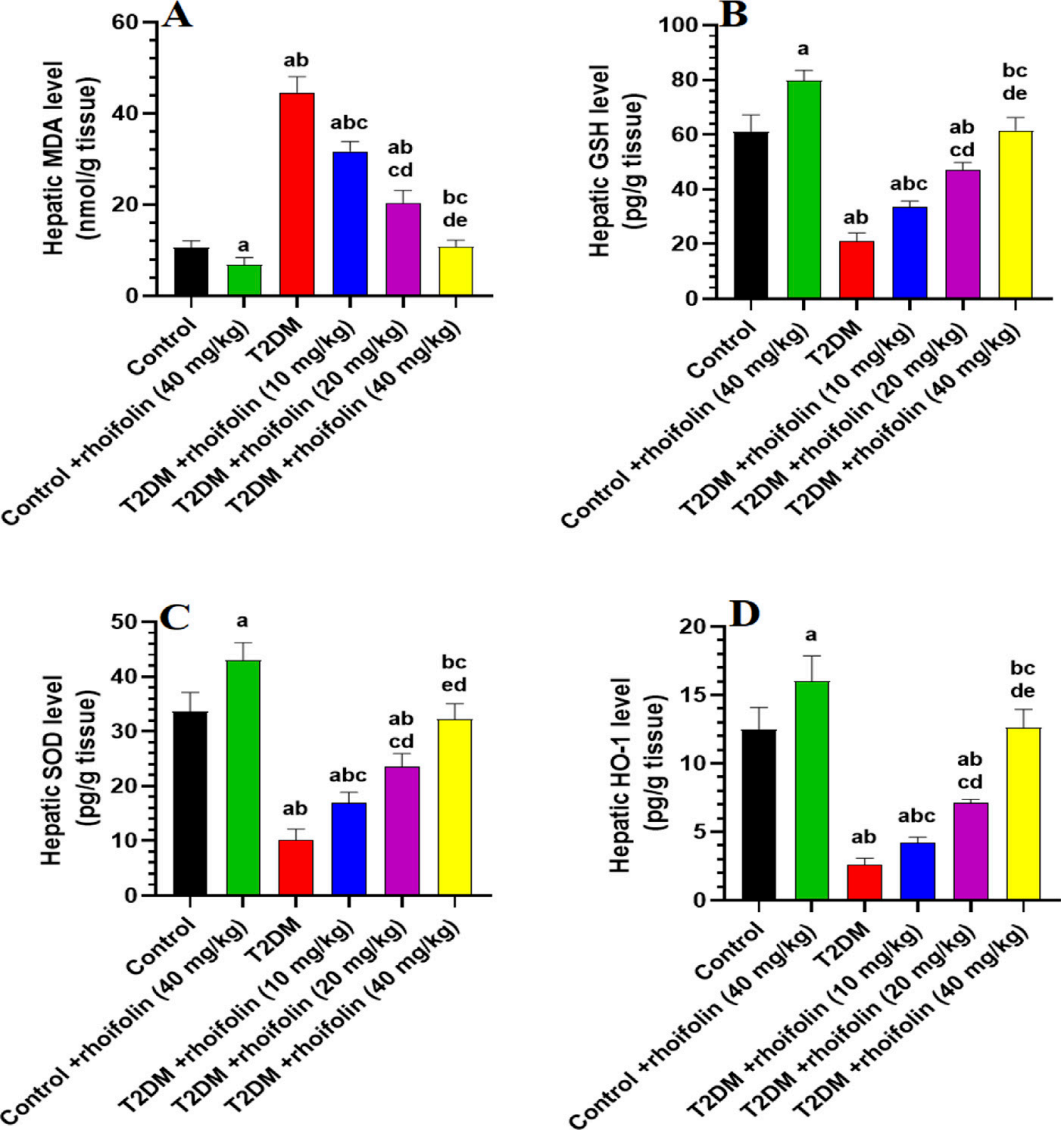
Analysis of hepatic biomarkers in experimental rat groups. **(A–D)**: illustrate the levels of malondialdehyde (MDA), glutathione (GSH), superoxide dismutase (SOD), and heme oxygenase-1 (HO-1) in the liver tissues of different rat groups. Data were analyzed by one-way ANOVA, and means were separated using the Tukey test at different significant levels (p < 0.05). Results are presented as means ± SD of seven samples per group. Statistical comparisons are denoted as follows: **(a)** *versus* the control group, **(b)** *versus* the control group treated with rhoifolin at 40 mg/kg, **(c)** *versus* the T2DM group, **(d)** *versus* the T2DM group treated with rhoifolin at 10 mg/kg, and **(e)** *versus* the T2DM group treated with rhoifolin at 20 mg/kg.

The levels of malondialdehyde (MDA), an index of lipid peroxidation, were significantly higher in the liver T2DM rats compared to control rats (p < 0.05). In T2DM rats’ livers, ROF treatment significantly reduced MDA levels dose-dependently. The most significant decrease occurred at the highest dose of 40 mg/kg (p < 0.05), which restored the MDA levels to those observed in the control rats. The control +40 mg/kg ROF rat group exhibited significantly reduced hepatic MDA levels compared to the control rat group (p < 0.05) (Figure 3A). Figures 3B–D demonstrates the levels of hepatic anti-oxidants GSH, SOD, and HO-1 in all experimental rat groups. The results showed that hepatic GSH, SOD, and HO-1 levels were significantly lower in T2DM rats than in control rats (p < 0.05). Generally, the levels of these markers significantly increased in T2DM rats following ROF treatment, depending on the dosage. The 40 mg/kg dose of ROF showed the best improvement in antioxidant levels, restoring the initial levels observed in the control group (p < 0.05). Also, the levels of GSH, SOD, and HO-1 in the liver were significantly higher in the control +40 mg/kg ROF group than in the control group (p < 0.05). These results show that ROF may help reduce oxidative stress in T2DM by enhancing the anti-oxidant activities of GSH, SOD, and HO-1 in the livers of both control and T2DM rats.

### 4.8 Effect of ROF on hepatic markers of apoptosis

**FIGURE 4:**
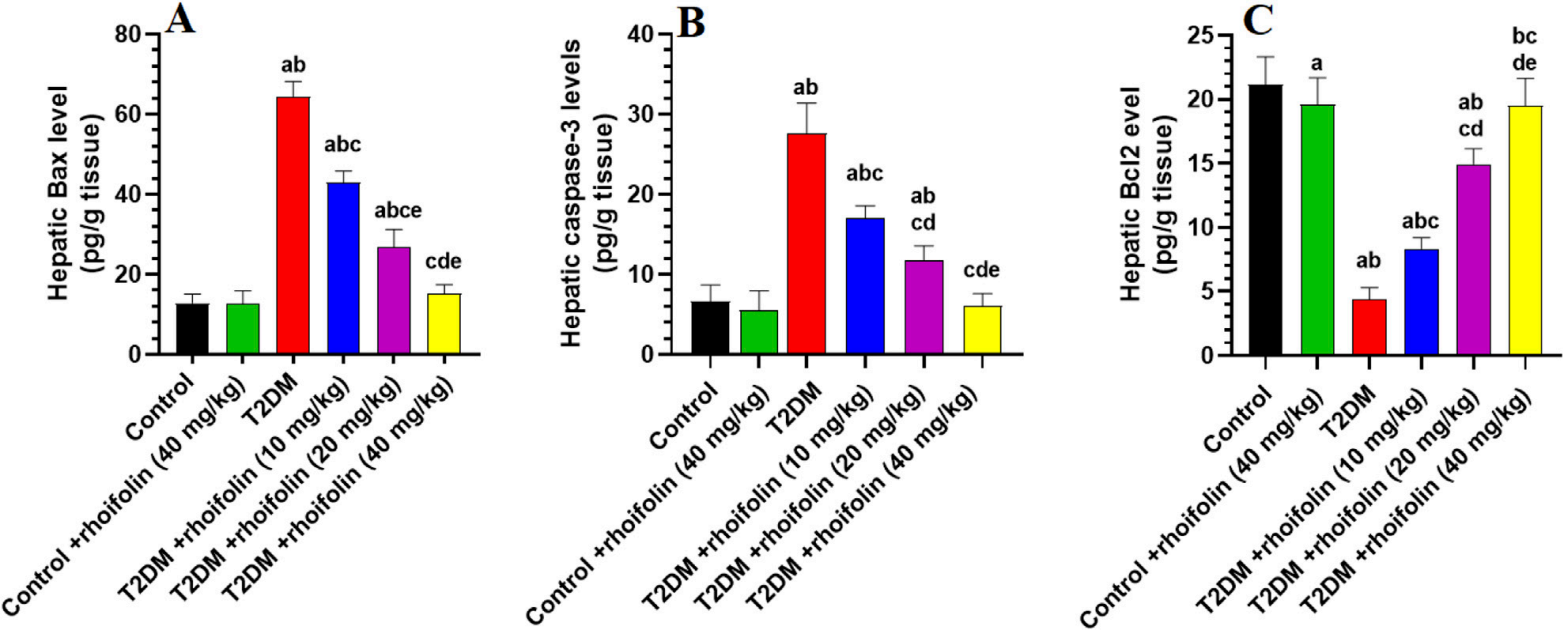
Hepatic apoptotic marker levels in experimental rat groups. The markers analyzed included Bax **(A)**, caspase-3 **(B)**, and Bcl2 **(C)**. Data were analyzed by one-way ANOVA, and means were separated using the Tukey test at different significant levels (p < 0.05). Results are presented as means ± SD of seven samples per group. Statistical comparisons are denoted as follows: **(a)** *versus* the control group, **(b)** *versus* the control treated with rhoifolin (40 mg/kg) group, **(c)** *versus* the T2DM group, **(d)** *versus* the T2DM group treated with rhoifolin (10 mg/kg), and **(e)** *versus* the T2DM group treated with rhoifolin (20 mg/kg).

Figures 4A–C displays the results of the apoptotic markers in the hepatocytes of all rat groups. The levels of Bax and caspase-3 were significantly increased, while the levels of Bcl2 were significantly reduced, in the livers of the T2DM rats compared to the control rats (p < 0.05).

In T2DM rats, ROF significantly modulated the hepatic apoptotic and anti-apoptotic markers. In more detail, treating rats with T2DM with ROF significantly lowered the elevated levels of Bax and caspase-3 in their livers. This effect varied with dose; the highest dose (40 mg/kg) produced the most significant impact on Bax and caspase-3 levels in T2DM rats, restoring these levels to those seen in control rats (p < 0.05) (Figures 4A,B). On the other hand, ROF doses significantly and gradually increased the low levels of Bcl2 in the livers of T2DM rats. The 40 mg/kg dose restored Bcl2 levels in T2DM rats to normal (p < 0.05). Besides, the hepatic Bcl-2 level in the control group was significantly higher than that in the control +40 mg/kg ROF group (p < 0.05) (Figure 4C). In contrast, the hepatic levels of Bax and caspase-3 in control rats did not significantly differ from those in the control +40 mg/kg ROF (Figures 4A,B).

### 4.9 Effect of ROF on expressions of hepatic mRNA and transcriptional activity markers

**FIGURE 5:**
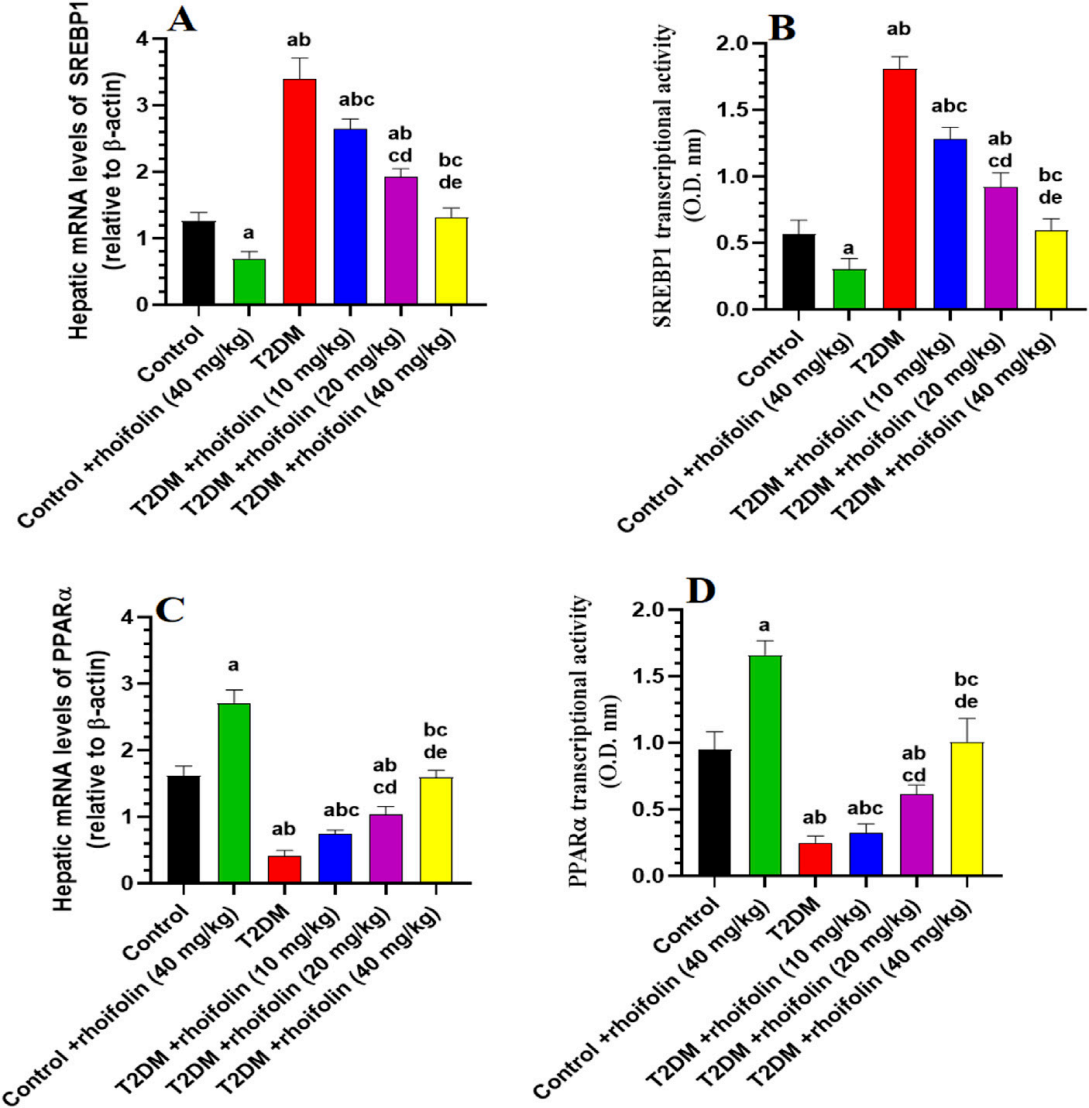
Hepatic mRNA expression and transcriptional activity of key metabolic regulators in experimental rat groups. This figure illustrates the hepatic mRNA levels and transcriptional activity of sterol regulatory element-binding protein 1 (SREBP1) **(A, B)** and peroxisome proliferator-activated receptor alpha (PPARα) **(C, D)** in various rat groups. Data were analyzed by one-way ANOVA, and means were separated using the Tukey test at different significant levels (p < 0.05). Results are presented as means ± SD of seven samples per group. Statistical significance is indicated as follows: **(a)** compared to the control group, **(b)** compared to the control treated with rhoifolin (40 mg/kg) group, **(c)** compared to the T2DM group, **(d)** compared to the T2DM group treated with rhoifolin (10 mg/kg), and **(e)** compared to the T2DM group treated with rhoifolin (20 mg/kg).

Figures 5A–D depicts the expression and transcriptional activity of mRNA SREBP-1 and PPARα in rat livers. Rats with T2DM had significantly higher levels of hepatic mRNA SREBP1 and lower levels of hepatic mRNA PPARα than the control rats (p < 0.05). Increasing doses of ROF significantly and gradually reduced the SREBP-1 level and transcriptional activity in the livers of rats with T2DM. At the 40 mg/kg dose, the level and transcriptional activity of this marker are comparable to those in the control rats (p < 0.05) (Figures 5A,B).

On the contrary, T2DM rats had significantly lower levels and transcriptional activity of mRNA PPARα than the control rats. The levels and the transcriptional activity of PPARα in the T2DM rats’ livers were significantly increased by applying ROF at different doses, especially at the 40 mg/kg dose (p < 0.05). This dosage elevated the activity and levels of PPARα in T2DM rats, yielding values comparable to the control group (p < 0.05) (Figures 5C,D).

The control +40 mg/kg ROF group exhibited a significantly reduced expression and transcriptional activity of mRNA SREBP1 compared to the control group (p < 0.05). Conversely, the expression and transcriptional activity of mRNA PPARα were significantly higher in the control +40 mg/kg ROF group compared to the control group (p < 0.05).

### 4.10 Effect of ROF on liver histology

**FIGURE 6:**
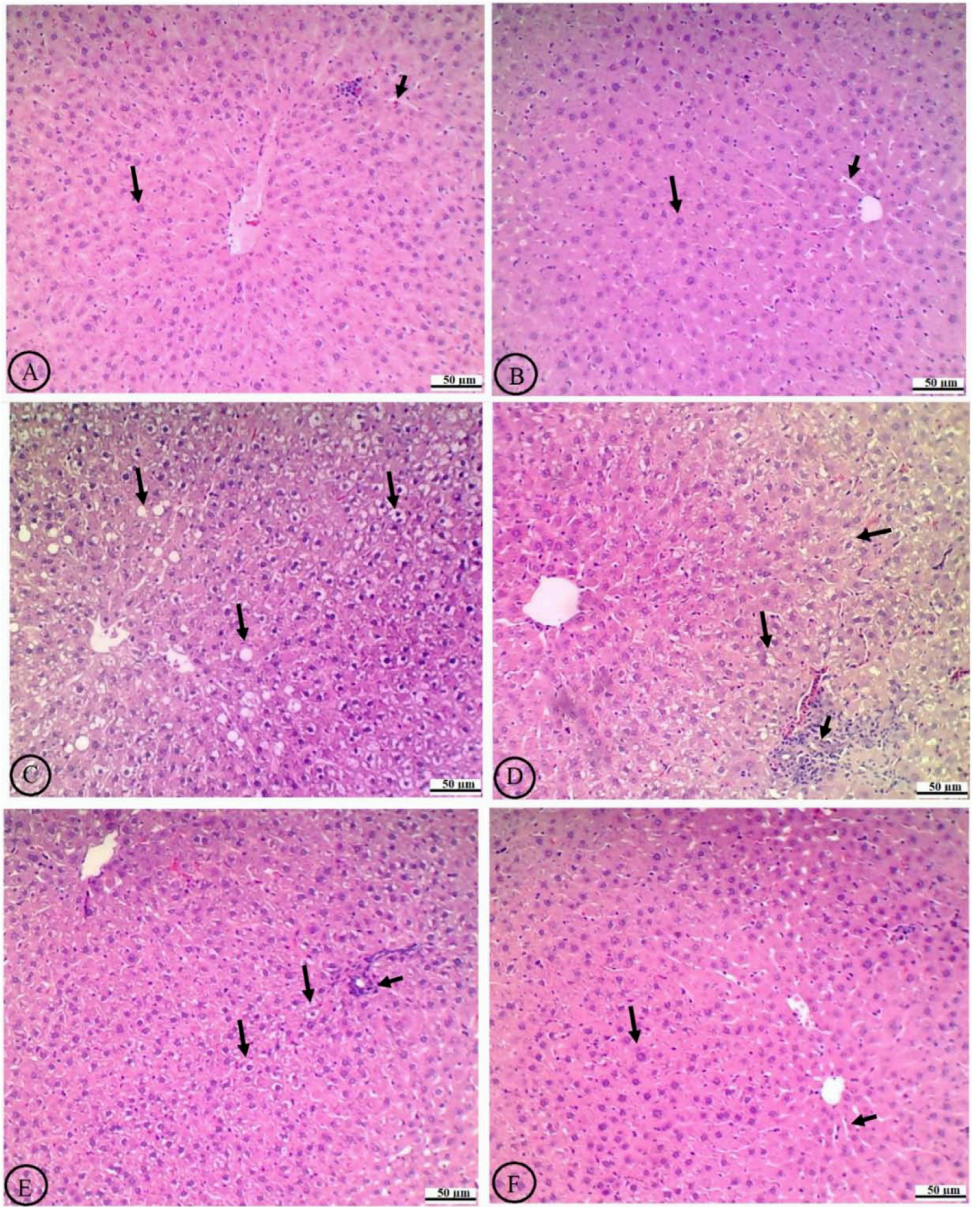
Histological analysis of liver tissue in experimental rat groups. The labels from **(A-F)** show variations in cellular structure and staining. Arrows indicate specific features like cell clusters or abnormalities. The scale bars measure fifty micrometers, providing a reference for size comparison. **(A)** The normal liver histology of the control rats treated with the vehicle shows hepatocytes with intact central veins (CV), normally sized sinusoids (short black arrow), and uniformly shaped and well-defined cell boundaries that have clear cytoplasm devoid of significant vacuolation (long black arrow). **(B)** The livers of control rats treated with rhoifolin (40 mg/kg) exhibit similar normal histological features. **(C)** The livers of T2DM rats exhibit marked histological alterations, characterized by enlarged hepatocytes, distorted cellular arrangement, and prominent, severe cytoplasmic vacuolation occupying most of the field, indicative of lipid accumulation (long black arrow). **(D)** Treating T2DM rats with rhoifolin at 10 mg/kg results in partial improvement, reduced vacuolation, and modest restoration of hepatocyte morphology (long black arrow). However, immune infiltration is dominant (short black arrow) **(E)** Treating the T2DM rats with rhoifolin at 20 mg/kg shows more pronounced improvements, with further reduction in the size and number of hepatocyte vacuoles (long lack arrow) and a more organized liver architecture. The number of infiltrated immune cells is also reduced (short black arrow). **(F)** Treating the T2DM rats with rhoifolin at 40 mg/kg achieves near-complete normalization of hepatocyte morphology (long black arrow) and sinusoids (short black arrow), as well as near-absence of cytoplasmic vacuolation, closely resembling normal liver tissue. This high-dose treatment demonstrates ROF’s robust efficacy in ameliorating T2DM-induced hepatic damage and steatosis.

In normal liver histology, hepatocytes typically exhibit a uniform shape with well-defined cell boundaries and a clear cytoplasm, devoid of significant vacuolation, were observed in the livers of the control rats treated with the vehicle or ROF (Figures 6A,B, respectively). In contrast, the liver tissue from T2DM rats displayed marked histological alterations, with hepatocytes that were enlarged, showed a distorted arrangement, and exhibited prominent and severe cytoplasmic vacuolation, indicative of lipid accumulation (Figure 6C).

Treatment with ROF led to a dose-dependent amelioration of these histological changes. At the lowest dose (10 mg/kg), the histological features of the liver showed partial improvement, including some reduction in vacuolation and a slight decline in liver fat (Figure 6D). The intermediate dose (20 mg/kg) resulted in more pronounced improvements, further reducing cytoplasmic vacuoles and a more organized liver architecture (Figure 6E). At the highest dose (40 mg/kg), hepatocyte morphology closely resembled that of normal liver tissue, with nearly complete normalization of cell shape and near-absence of cytoplasmic vacuolation (Figure 6F). This high-dose treatment effectively restored the liver’s histological architecture to a near-normal state, demonstrating ROF’s robust efficacy in mitigating T2DM-induced hepatic damage and steatosis.

## 5 Discussion

This study provides decisive evidence that ROF offers substantial protection against diabetic liver damage in T2DM rats, demonstrating a dose-dependent therapeutic potential. ROF effectively ameliorates T2DM-induced hepatic damage through multifaceted mechanisms, including hypoglycemic, hypolipidemic, anti-oxidant, and anti-inflammatory actions. It enhances insulin sensitivity, reduces liver injury and steatosis, and inhibits key hepatic enzymes and transcription factors such as G6Pase, FBP-1, and SREBP1, which are crucial in gluconeogenesis and lipogenesis. The ROF ability to modulate oxidative stress is demonstrated by its restoration of anti-oxidant levels (SOD, GSH) and suppression of NF-κB activation and inflammatory cytokines (TNF-α, IL-6) in diabetic livers. Additionally, the compound’s protective effects also extend to control rats, highlighting its potential as a preventive therapy against T2DM by targeting these critical pathways. These findings align with previous studies demonstrating ROF’s capacity to enhance anti-oxidant defenses and mitigate oxidative stress across various models, including cisplatin-induced lung damage, testicular damage, and other conditions (Eldahshan and Azab, 2012; El-Shawi and Eldahshan, 2014; Peng et al., 2020a; Zhang et al., 2021b; Mai et al., 2022a; Saher et al., 2023; Akbar et al., 2024).

Insulin regulates lipogenesis in adipose tissue, glycogen synthesis in the liver and muscles, while inhibiting hepatic gluconeogenesis (Zhang et al., 2023). Insulin resistance (IR), a significant factor in T2DM, disrupts glucose and lipid homeostasis (Lu et al., 2019). In our study, we used HFD to induce obesity, followed by a single low-dose intraperitoneal injection of STZ to induce T2DM and hepatic steatosis in rats, which were then involved in a designed ROF treatment experiment lasting 8 weeks. This model replicates T2DM and associated organ damage, reflecting features of human disease with minimal mortality and no requirement for external insulin (Galicia-Garcia et al., 2020). The model demonstrates how diet-induced metabolic disturbances and impaired β-cell function led to IR (Lu et al., 2019; Zhang et al., 2024). HFD causes obesity, increases inflammatory cytokines (e.g., TNF-α, IL-6), and promotes lipid accumulation in the liver, which impairs insulin receptor function (Zhang et al., 2023). STZ exacerbates IR by destroying pancreatic beta cells, which reduces insulin production and triggers systemic inflammation. This inflammation, accompanied by an influx of increased free fatty acids, leads to mitochondrial dysfunction, lipid accumulation, impaired glucose metabolism, hyperglycemia, and dyslipidemia (Gao et al., 2023; Yang and Wang, 2023). Weight loss in this model is driven by increased energy expenditure, reduced food intake efficiency, and muscle wasting (Ravussin et al., 2021). In this context, IR may impede the translocation of the GLUT4 receptor to the skeletal muscle membrane, thereby hindering glucose uptake by skeletal muscles. As a result, muscular glycogen stores are reduced. This insufficient energy storage in skeletal muscle contributes to muscle wasting and sarcopenia in individuals with T2DM, leading to weight loss (Bhat and Mani, 2023). Elevated basal metabolic rates and ineffective glucose utilization further exacerbate fat and muscle breakdown (McPherron et al., 2013; Zhang et al., 2021b). Additionally, weight loss may occur due to dehydration and osmotic diuresis resulting from hyperglycemia (Li et al., 2016).

In this research, we observed a decrease in food consumption, reduced body weight, fat weight, BMI, increased IR, and dyslipidemia characterized by hypercholesterolemia and hypertriglyceridemia, hepatic steatosis, and hepatomegaly in T2DM rat model, which validates our animal model and support many previous studies (Vornoli et al., 2014; Galicia-Garcia et al., 2020; Song et al., 2020). Conversely, the decline in food consumption noted in T2DM rats is likely due to the pathogenic effects of diabetes, which can lead to gastroparesis or gastrointestinal distress, presenting as early satiety, nausea, vomiting, and stomach pain. All these factors may contribute to a loss of appetite, ultimately resulting in reduced caloric intake (CDC, 2025). However, all these pathological and biochemical features in T2DM rats were primarily ameliorated following treatment with ROF. This illustrates ROF’s protective potential against obesity and T2DM. Interestingly, treatment with ROF did not alter glucose or insulin levels, nor HOMA-IR in control rats, suggesting that this drug’s insulin-sensitizing effect mainly occurs in diabetic conditions. In addition, the antidiabetic effects of ROF were independent of food intake, as no reduction in food intake was observed between the diabetic control rats and those administered three doses of ROF. Similarly, treatment with the highest dose of ROF did not affect food intake in control rats. This aligns with the study by Jung et al. (2016), which illustrated that apigenin extract reduces body weight loss and IR in HFD-fed rats without affecting appetite or food intake. Although the anti-diabetic effects of ROF have not yet been characterized in animals or humans with metabolic disturbances, previous *in vitro* studies have demonstrated ROF’s ability to mimic insulin action, even at a molar concentration (0.01–5 μM) where it exhibited an equal potential to insulin for phosphorylating insulin receptor substrate (IRS) and enhancing the membrane expression of GLUT-4 in differentiated 3T3-L1 adipocytes (Rao et al., 2011b). Ehsan et al. (2025) demonstrated that ROF appreciably upregulated the expression of GLUT-4, thereby improving insulin sensitivity. Other authors have shown that ROF can inhibit amylase, potentially reducing intestinal glucose absorption (Zengin et al., 2023b). In the same vein, since we have observed low serum levels of TNF-α, IL-6, and FFAs in the control and T2DM rats, we suggest that ROF attenuates peripheral IR and related complications in these diabetic rats by suppressing adipose tissue inflammation, as highlighted by previous studies (Peng et al., 2020a; Mai et al., 2022a). However, our data still makes it unclear whether ROF acts through other mechanisms, such as modulating adipogenesis and fatty acid oxidation in adipose tissue, which requires further studies targeting adipose tissue.

In diabetic animals, glucose and lipid homeostasis are severely disrupted, leading to persistent hyperglycemia and hyperlipidemia, which are primary contributors to multi-organ damage and atherosclerosis (Bhat and Mani, 2023). The transcription factor SREBP1c plays a crucial role in hepatic lipid synthesis by upregulating key lipogenic genes, including acetyl-CoA carboxylase (ACC) and fatty acid synthase (Moslehi and Hamidi-Zad, 2018). In a healthy liver, this is countered by PPARα, which promotes mitochondrial FA transport and oxidation (Qiu et al., 2023). However, IR in adipose tissue enhances the influx of FFA into the liver, further activating SREBP1c and driving excessive lipid synthesis, thus aggravating hepatic steatosis, oxidative stress, and inflammation (Yang and Wang, 2023; Zhang et al., 2024). Elevated glucose levels, ROS, endoplasmic reticulum stress, and inflammatory cytokines also contribute to SREBP1c activation in obesity and T2DM (Sekiya et al., 2008; Uttarwar et al., 2012; Kusnadi et al., 2019; Zhou et al., 2019). In contrast, liver tissues from HFD rodents with T2DM exhibit reduced PPARα expression and elevated SREBP1c levels (Moslehi and Hamidi-Zad, 2018; Weber et al., 2020; Badmus et al., 2022). Enhancing PPARα activity or inhibiting SREBP1c are effective strategies to mitigate liver steatosis and damage in obese and diabetic animals (Moslehi and Hamidi-Zad, 2018; Kusnadi et al., 2019; Zhou et al., 2019). Furthermore, impaired hepatic insulin-signaling leads to increased gluconeogenesis and decreased glycogenolysis, along with elevated levels of gluconeogenic enzymes such as G-6-Pase and FBP-1 and reduced glycolytic enzymes like hexokinase and glucokinase in the livers of diabetic animals (Hatting et al., 2018; Song et al., 2020).

In this investigation, we observed the remarkable potential of ROF to mitigate hepatic lipogenesis in the livers of diabetic rats. Indeed, ROF treatment significantly reduced hepatic mRNA expression levels of SREBP1 while stimulating those of PPARα in the livers of both control and T2DM rats. In this regard, treatment with ROF lowered serum and hepatic levels of TGs and CHOL and decreased hepatic levels of LDL-c in both control and T2DM rats. These effects suggest that ROF has hypolipidemic properties due to its independent ability to stimulate PPARα and downregulate SREBP1. However, ROF only reduced serum and hepatic levels of FFAs in rats with T2DM. This may be attributed to the previously discussed improvement in insulin signaling and the suppression of adipose tissue lipolysis. Additionally, ROF reduced the levels of G-6-Pase in the livers of T2DM rats, while increasing the levels of glucokinase; however, no significant changes were observed in these enzymes in the livers of control rats. This might explain the previously mentioned lack of effect of ROF treatment on fasting glucose levels. Therefore, we suggest that ROF’s hypoglycemic and regulatory impact on glucose metabolism-related enzymes in the livers of T2DM individuals results from improved IR. IR in T2DM is primarily associated with low-grade, tissue-specific inflammatory responses induced by various pro-inflammatory and oxidative stress mediators, notably pro-inflammatory cytokines such as interleukin-1 beta (IL-1β), interleukin-6 (IL-6), tumor necrosis factor-alpha (TNF-α), and numerous chemokines and adipocytokines (e.g., leptin and resistin) (Rehman and Akash, 2016). Additionally, insulin resistance affects various immune cells, including T cells, B cells, macrophages, and neutrophils, leading to an imbalance between pro-inflammatory and anti-inflammatory responses (Berbudi et al., 2025). For example, macrophages shift from an anti-inflammatory M2 to a pro-inflammatory M1 phenotype, releasing cytokines such as TNF-α, IL-6, and IL-1β that impair insulin signaling in adipose tissue, liver, and muscle (Berbudi et al., 2025; Hameed et al., 2015). Furthermore, T lymphocytes exhibit a metabolic shift toward fatty acid oxidation, which increases the number of pro-inflammatory Th17 cells while reducing the number of regulatory T cells (Tregs). This shift exacerbates systemic inflammation and insulin resistance (Berbudi et al., 2025; Nordmann et al., 2017). Consequently, elevated pro-inflammatory cytokines and adipokines hinder insulin signaling, creating a vicious cycle of metabolic and immune dysregulation (Berbudi et al., 2025). These pro-inflammatory cytokines, particularly TNF-α and IL-6, can lead to insulin resistance in adipose tissue, skeletal muscle, and the liver by disrupting insulin signaling. This disruption occurs through promoting serine phosphorylation (instead of tyrosine) in insulin receptor substrates (IRS-1/2), which impairs GLUT4 function and reduces insulin sensitivity (de Luca and Olefsky, 2008; Hoca, 2023; Shoelson et al., 2006). These cytokines reduce adiponectin, an insulin-sensitizing hormone, and promote lipolysis, releasing free fatty acids that exacerbate insulin resistance (Dandona et al., 2004; Hoca, 2023). Increased circulating free fatty acids can lead to decreased insulin sensitivity due to an increase in intracellular lipid products, including fatty acyl-CoA and ceramide. These lipid intermediates can activate the serine/threonine kinase, protein kinase C-θ (PKCθ), inhibiting the insulin signaling cascade. Additionally, saturated FFAs activate Toll-like receptor 4 (TLR4) and nuclear factor kappa B (NF-κB), leading to the expression of pro-inflammatory cytokines such as TNF-α and IL-6 (Hotamisligil, 2006).

Chronic inflammation disrupts protein folding in the ER, activating pathways that worsen insulin resistance and β-cell apoptosis (Hameed et al., 2015), while damaging peripheral tissues through increased ROS, impairing mitochondrial function, and activating stress kinases and pro-inflammatory signaling pathways (Brownlee, 2005a; Hotamisligil, 2006; Hoca, 2023) that further inhibit insulin signaling (Hoca, 2023) in muscles and the liver (Hameed et al., 2015). Perturbations in glucose metabolism due to insulin resistance are exacerbated when insulin production is compromised, as seen in patients with T2DM (de Luca and Olefsky, 2008). On one hand, hyperglycemia facilitates the formation of AGEs, which further amplify oxidative stress by enhancing ROS generation and inflammatory responses (Brownlee, 2005a). On the other hand, the polyol pathway, activated by elevated glucose levels, converts excess glucose into sorbitol through aldose reductase, thereby depleting NADPH and exacerbating oxidative stress (Tang et al., 2012). Concurrently, protein kinase C (PKC), particularly PKCβ, is activated by diacylglycerol (DAG), enhancing NADPH oxidase activity and ROS production (Cenni et al., 2002). NADPH oxidase-generated ROS react with nitric oxide to form peroxynitrite, exacerbating cellular injury (Miller et al., 2009). Additionally, elevated blood glucose and FFAs in T2DM activate the NLRP3 inflammasome, triggering IL-1β and IL-18 release. These cytokines promote inflammation and directly interfere with insulin receptor signaling (Hameed et al., 2015; Lu et al., 2023). Pro-inflammatory cytokines like IL-1β and TNF-α cause β-cell dedifferentiation (loss of insulin-producing identity) and apoptosis. IL-1β is particularly potent, reducing insulin secretion capacity and promoting β-cell failure (Lu et al., 2023; Nordmann et al., 2017). As β-cells fail, insulin secretion declines, exacerbating hyperglycemia and metabolic stress, which in turn activates more immune cells and inflammatory pathways (Lu et al., 2023; Nordmann et al., 2017). This persistent oxidative stress and inflammation disrupt insulin signaling, perpetuating a cycle of ROS production and liver dysfunction by activating the Kupffer cells, increasing macrophage infiltration, damaging the mitochondria, activating lipogenesis, and promoting endoplasmic reticulum stress (Al-Harbi et al., 2021). Furthermore, these ROS and inflammatory cytokines contribute to hepatic IR, inducing liver necrosis and apoptosis by promoting membrane lipid peroxidation, DNA damage, and the activation of the P53/Bax apoptotic axis (Redza-Dutordoir and Averill-Bates, 2016).

In this study, we observed that T2DM rats exhibited elevated levels of lipid peroxides (MDA), IL-6, TNF-α, Bax, and caspase-3, as well as increased NF-κB mRNA and nuclear expression, reflecting severe oxidative stress, inflammation, and apoptosis. Concurrently, the levels of Bcl2 and key antioxidants such as GSH, SOD, and HO-1 were significantly depleted, highlighting a disrupted antioxidant defense system and reduced anti-apoptotic response. Mitochondrial dysfunction and inflammatory signaling lead to elevated ROS levels, driving NF-κB activation, exacerbating inflammation, and inducing cellular apoptosis by lowering Bcl2 levels and upregulating Bax (Sun et al., 2023; Ouyang et al., 2024). Notably, ROF was successfully tested at all doses and progressively reduced these effects in a dose-dependent manner. It restored antioxidant levels in both control and T2DM rats and significantly suppressed NF-κB activation, inflammatory cytokine production, and Bax activation, specifically in the livers of T2DM rats. This suggests that ROF’s mechanism enhances antioxidant defenses, likely reducing NF-κB activation, inflammation, and apoptosis. Furthermore, ROF has demonstrated significant effects in reducing IR, as evidenced by lowered HOMA-IR and enhanced glycemic control; thus, improvements in insulin sensitivity may occur. Connecting the impact of ROF to inflammation and cell damage involves several processes at the molecular and cellular levels, operating through anti-inflammatory and antioxidant actions. Our findings are supported by numerous experimental studies that demonstrate similar effects in other animal models. For instance, ROF at a dose of 10 mg/kg of body weight attenuated cisplatin-mediated lung damage by enhancing levels of GSH and other antioxidant enzymes such as SOD, CAT, glutathione S-transferase (GST), glutathione reductase (GRx), and glutathione peroxidase (GPx) while decreasing lipid peroxidation, the activation of NF-κB, IL-6, Bax, caspase-3, and caspase 9 (Akbar et al., 2024). It also inhibited cisplatin from causing testicular damage and infertility in rats through typical mechanisms (Saher et al., 2023). ROF at doses of 2.5, 2.5, and 250 mg/kg significantly inhibited rat paw edema by reducing prostaglandin E2 and TNF-α levels while increasing total antioxidant capacity in a rat model of experimental carrageenan (Eldahshan and Azab, 2012). It also prevented cardiac damage and dysfunction in rats exposed to gamma irradiation by stimulating GSH and SOD while attenuating MDA levels (El-Shawi and Eldahshan, 2014). Furthermore, ROF prevented damage to the hippocampus and subsequent memory loss in a rat model of Alzheimer’s disease induced by STZ by reducing MDA levels and increasing the levels of GSH, SOD, GPx, and GRx (Zhang et al., 2021b). In a rat model of Freund’s adjuvant-induced rheumatoid arthritis, ROF’s protective effect primarily involved inhibiting NF-kB and suppressing cytokine production (Peng et al., 2020a). Further studies have shown ROF’s ability to suppress alcoholic fatty liver disease by maintaining the antioxidant balance in hepatocytes, upregulating GSH and SOD, downregulating CYP2E1 and TLR4, and decreasing the phosphorylation of NF-κB (Mai et al., 2022a). It has been reported that ROF at doses of 10 and 20 mg/kg of body weight improves glycometabolic control and protects against β-cell dysfunction by upregulating the expression of insulin signaling proteins (PDX-1, INS-1, GLUT-4), which are essential for β-cell survival and insulin production, promoting metabolic homeostasis. It also downregulates the MAPK/JNK pathway, which prevents stress-induced apoptosis and IRS-1 serine phosphorylation, thus preserving insulin signaling (Ehsan et al., 2025). In conclusion, ROF helps improve IR in T2DM by reducing inflammation, lowering oxidative stress, boosting antioxidant levels, modulating dyslipidemia, and enhancing the expression of genes related to insulin signaling. These effects collectively improve glycemic control, protect cells from inflammation-induced injury, and restore insulin sensitivity, highlighting its potential as a therapeutic agent. However, further studies are required to confirm this mechanism and its therapeutic implications.

## 6 Conclusion

Our study strongly indicates that ROF significantly alleviates diabetic liver damage through its hypoglycemic, hypolipidemic, anti-oxidant, and anti-inflammatory properties. It enhances anti-oxidant defenses and suppresses pro-inflammatory pathways, particularly by inhibiting NF-κB and upregulating endogenous anti-oxidants. Therefore, ROF presents a promising therapeutic strategy for T2DM and its complications. These findings highlight ROF’s potential as a novel intervention for T2DM, warranting further clinical exploration to validate its therapeutic efficacy and safety.

## 7 Limitations and future directions

This study, while demonstrating the significant protective effects of ROF against diabetic liver damage, does have limitations that warrant consideration. First, the study primarily utilized a rat model, which, although relevant, may not completely replicate human metabolic and pathological conditions, potentially limiting the translational applicability of the findings. Secondly, while the study highlights the impact of ROF on key antioxidant and inflammatory pathways, it does not provide a comprehensive mechanistic understanding of its interactions with other metabolic pathways and signaling networks. Additionally, the effects of ROF on long-term outcomes and its safety profile with chronic use remain unexplored. Future research should focus on several key areas. Extensive studies are needed to elucidate the precise molecular mechanisms underlying ROF’s modulation of the Nrf2 pathway and its interaction with other antioxidant and inflammatory pathways. Clinical trials are essential for validating the efficacy and safety of ROF in human subjects, thereby bridging the translational gap between animal models and clinical applications. Investigations on the impact of ROF on additional metabolic disturbances and its long-term effects will provide a clearer picture of its therapeutic potential. Furthermore, exploring the ROF’s impact on other organs affected by diabetes and its interactions with conventional diabetes therapies could offer insights into its broader utility and potential as a complementary treatment.

## Data Availability

The original contributions presented in the study are included in the article/supplementary material, further inquiries can be directed to the corresponding authors.

## References

[B1] Adebayo A. O. Akinloye A. K. Oke B. O. Taiwo V. O. (2020). Relationship between body mass index (BMI) and testicular and hormonal parameters of sexually active Male greater cane rats (Thryonomys swinderianus). Anim. Reprod. 17, e20190026. 10.21451/1984-3143-AR2019-0026 32368277 PMC7189494

[B2] Akbar A. Azmat R. Batool M. Almutairi B. Riaz M. (2024). Rhoifolin protects cisplatin mediated pulmonary toxicity via attenuation of oxidative stress, inflammatory response, apoptosis and histopathological damages. J. King Saud Univ. - Sci. 36, 103149. 10.1016/j.jksus.2024.103149

[B3] Al-Harbi L. N. Alshammari G. M. Al-Dossari A. M. Subash-Babu P. Binobead M. A. Alhussain M. H. (2021). Beta vulgaris L.(Beetroot) methanolic extract prevents hepatic steatosis and liver damage in T2DM rats by hypoglycemic, insulin-sensitizing, antioxidant effects, and upregulation of PPARα. Biology 10, 1306. 10.3390/biology10121306 34943221 PMC8698622

[B4] Al-Shalabi E. Abusulieh S. Hammad A. M. Sunoqrot S. (2022). Rhoifolin loaded in PLGA nanoparticles alleviates oxidative stress and inflammation *in vitro* and *in vivo* . Biomaterials Sci. 10, 5504–5519. 10.1039/d2bm00309k 35920694

[B5] Arika W. M. Kibiti C. M. Njagi J. M. Ngugi M. P. (2019). Anti-obesity effects of dichloromethane leaf extract of Gnidia glauca in high fat diet-induced Obese rats. Heliyon 5, e02800. 10.1016/j.heliyon.2019.e02800 31844729 PMC6895710

[B6] Badmus O. O. Hillhouse S. A. Anderson C. D. Hinds T. D. Stec D. E. (2022). Molecular mechanisms of metabolic associated fatty liver disease (MAFLD): functional analysis of lipid metabolism pathways. Clin. Sci. (Lond) 136, 1347–1366. 10.1042/CS20220572 36148775 PMC9508552

[B7] Bancroft J. D. Gamble M. (2008). Theory and practice of histological techniques. 6th edition. Churchill Livingstone Elsevier, 126–127.

[B8] Berbudi A. Khairani S. Tjahjadi A. I. (2025). Interplay between insulin resistance and immune dysregulation in type 2 diabetes mellitus: implications for therapeutic interventions. ImmunoTargets Ther. 14, 359–382. 10.2147/ITT.S499605 40196377 PMC11974557

[B9] Bhat N. Mani A. (2023). Dysregulation of lipid and glucose metabolism in nonalcoholic fatty liver disease. Nutrients 15, 2323. 10.3390/nu15102323 37242206 PMC10222271

[B10] Bligh E. G. Dyer W. J. (1959). A rapid method of total lipid extraction and purification. Can. J. Biochem. Physiol. 37, 911–917. 10.1139/o59-099 13671378

[B11] Brownlee M. (2005a). The pathobiology of diabetic complications: a unifying mechanism. Diabetes 54, 1615–1625. 15919781 10.2337/diabetes.54.6.1615

[B12] Brownlee M. (2005b). The pathobiology of diabetic complications: a unifying mechanism. diabetes 54, 1615–1625. 10.2337/diabetes.54.6.1615 15919781

[B13] Bustin S. A. Benes V. Garson J. A. Hellemans J. Huggett J. Kubista M. (2009). The MIQE guidelines: minimum information for publication of quantitative real-time PCR experiments. Clin. Chem. 55, 611–622. 10.1373/clinchem.2008.112797 19246619

[B14] Cazarolli L. H. Folador P. Moresco H. H. Brighente I. M. C. Pizzolatti M. G. Silva F. R. M. B. (2009). Stimulatory effect of apigenin-6-C-β-L-fucopyranoside on insulin secretion and glycogen synthesis. Eur. J. Med. Chem. 44, 4668–4673. 10.1016/j.ejmech.2009.07.001 19625113

[B15] CDC (2025). Centers for disease control and prevention. Diabetes. Available online at: https://www.cdc.gov/diabetes/diabetes-complications/diabetes-and-digestion.html.

[B16] Cenni V. Döppler H. Sonnenburg E. D. Maraldi N. Newton A. C. Toker A. (2002). Regulation of novel protein kinase C epsilon by phosphorylation. Biochem. J. 363, 537–545. 10.1042/0264-6021:3630537 11964154 PMC1222506

[B17] Chen X. Song M. Zhang B. Zhang Y. (2016). Reactive oxygen species regulate T cell immune response in the tumor microenvironment. Oxidative Med. Cell. Longev. 2016, 1580967. 10.1155/2016/1580967 PMC4980531 27547291

[B18] Cicero A. F. Baggioni A. (2016). Berberine and its role in chronic disease. Anti-inflammatory nutraceuticals chronic Dis. 928, 27–45. 10.1007/978-3-319-41334-1_2 27671811

[B19] Dandona P. Aljada A. Bandyopadhyay A. (2004). Inflammation: the link between insulin resistance, obesity and diabetes. Trends Immunol. 25, 4–7. 10.1016/j.it.2003.10.013 14698276

[B20] De Luca C. Olefsky J. M. (2008). Inflammation and insulin resistance. FEBS Lett. 582, 97–105. 10.1016/j.febslet.2007.11.057 18053812 PMC2246086

[B21] Ehsan M. Ahmed S. Majeed W. Iftikhar A. Iftikhar M. Abbas M. (2025). Rhoifolin improves glycometabolic control in streptozotocin-induced diabetic rats by Up-Regulating the expression of insulin signaling proteins and down-regulating the MAPK/JNK pathway. Pharmaceuticals 18, 361. 10.3390/ph18030361 40143138 PMC11944882

[B22] Eldahshan O. Azab S. (2012). Anti-inflammatory effect of Apigenin-7-neohesperidoside (rhoifolin) in carrageenin-induced rat oedema model. J. Appl. Pharm. Sci. 2, 74–79. 10.7324/JAPS.2012.2811

[B24] El-Shawi O. E. Eldahshan O. A. (2014). Protective effect of rhoifolin on gamma irradiation induced cardiac dysfunctions in albino mice. Arab. J. Nucl. Sci. Appl. 47, 198–207.

[B25] Farrag E. A. Hammad M. O. Safwat S. M. Hamed S. Hellal D. (2023). Artemisinin attenuates type 2 diabetic cardiomyopathy in rats through modulation of AGE-RAGE/HMGB-1 signaling pathway. Sci. Rep. 13, 11043. 10.1038/s41598-023-37678-w 37422477 PMC10329689

[B26] Galicia-Garcia U. Benito-Vicente A. Jebari S. Larrea-Sebal A. Siddiqi H. Uribe K. B. (2020). Pathophysiology of type 2 diabetes mellitus. Int. J. Mol. Sci. 21, 6275. 10.3390/ijms21176275 32872570 PMC7503727

[B27] Gao Y. M. Chen W. J. Deng Z. L. Shang Z. Wang Y. (2023). Association between triglyceride-glucose index and risk of end-stage renal disease in patients with type 2 diabetes mellitus and chronic kidney disease. Front. Endocrinol. (Lausanne) 14, 1150980. 10.3389/fendo.2023.1150980 37152938 PMC10157287

[B28] Godoy-Matos A. F. Silva Júnior W. S. Valerio C. M. (2020). NAFLD as a continuum: from obesity to metabolic syndrome and diabetes. Diabetology & metabolic syndrome 12, 60–20. 10.1186/s13098-020-00570-y 32684985 PMC7359287

[B29] González P. Lozano P. Ros G. Solano F. (2023). Hyperglycemia and oxidative stress: an integral, updated and critical overview of their metabolic interconnections. Int. J. Mol. Sci. 24, 9352. 10.3390/ijms24119352 37298303 PMC10253853

[B30] Gregor M. F. Hotamisligil G. S. (2011). Inflammatory mechanisms in obesity. Annu. Rev. Immunol. 29, 415–445. 10.1146/annurev-immunol-031210-101322 21219177

[B31] Hameed I. Masoodi S. R. Mir S. A. Nabi M. Ghazanfar K. Ganai B. A. (2015). Type 2 diabetes mellitus: from a metabolic disorder to an inflammatory condition. World J. diabetes 6, 598–612. 10.4239/wjd.v6.i4.598 25987957 PMC4434080

[B32] Hao M. Lv Y. Liu S. Guo W. (2024). The new challenge of obesity-obesity-associated nephropathy. Diabetes, Metabolic Syndrome Obes. 17, 1957–1971. 10.2147/DMSO.S433649 PMC11086398 38737387

[B33] Hatting M. Tavares C. D. J. Sharabi K. Rines A. K. Puigserver P. (2018). Insulin regulation of gluconeogenesis. Ann. N. Y. Acad. Sci. 1411, 21–35. 10.1111/nyas.13435 28868790 PMC5927596

[B34] Hoca M. (2023). The reciprocal relationship between inflammation and diabetes: importance of medical nutrition therapy. cjms. 8, 166–172. 10.4274/cjms.2023.2022-60

[B35] Hotamisligil G. S. (2006). Inflammation and metabolic disorders. Nature 444, 860–867. 10.1038/nature05485 17167474

[B36] Jha D. Bakker E. N. Kumar R. (2024). Mechanistic and therapeutic role of NLRP3 inflammasome in the pathogenesis of alzheimer's disease. J. Neurochem. 168, 3574–3598. 10.1111/jnc.15788 36802053

[B37] Jung U. J. Cho Y. Y. Choi M. S. (2016). Apigenin ameliorates dyslipidemia, hepatic steatosis and insulin resistance by modulating metabolic and transcriptional profiles in the liver of high-fat diet-induced Obese mice. Nutrients 8, 305. 10.3390/nu8050305 27213439 PMC4882717

[B38] Kang O.-H. Lee J.-H. Kwon D.-Y. (2011). Apigenin inhibits release of inflammatory mediators by blocking the NF-κB activation pathways in the HMC-1 cells. Immunopharmacol. Immunotoxicol. 33, 473–479. 10.3109/08923973.2010.538851 21142820

[B40] Kosmas C. E. Silverio D. Tsomidou C. Salcedo M. D. Montan P. D. Guzman E. (2018). The impact of insulin resistance and chronic kidney disease on inflammation and cardiovascular disease. Clin. Med. Insights Endocrinol. Diabetes 11, 1179551418792257. 10.1177/1179551418792257 30083062 PMC6071166

[B41] Kusnadi A. Park S. H. Yuan R. Pannellini T. Giannopoulou E. Oliver D. (2019). The cytokine TNF promotes transcription factor SREBP activity and binding to inflammatory genes to activate macrophages and limit tissue repair. Immunity 51, 241–257. 10.1016/j.immuni.2019.06.005 31303399 PMC6709581

[B42] Li F. Lei T. Xie K. Wu X. Tang C. Jiang M. (2016). Effects of extremely low frequency pulsed magnetic fields on diabetic nephropathy in streptozotocin-treated rats. Biomed. Eng. online 15, 8–13. 10.1186/s12938-015-0121-6 26786255 PMC4717615

[B43] Li Y. Cheng Y. Zhou Y. Du H. Zhang C. Zhao Z. (2022). High fat diet-induced obesity leads to depressive and anxiety-like behaviors in mice *via* AMPK/mTOR-mediated autophagy. Exp. Neurol. 348, 113949. 10.1016/j.expneurol.2021.113949 34902357

[B44] Liao S. Song F. Feng W. Ding X. Yao J. Song H. (2019). Rhoifolin ameliorates titanium particle‐stimulated osteolysis and attenuates osteoclastogenesis *via* RANKL‐induced NF‐κB and MAPK pathways. J. Cell. Physiology 234, 17600–17611. 10.1002/jcp.28384 30854667

[B45] Livak K. J. Schmittgen T. D. (2001). Analysis of relative gene expression data using real-time quantitative PCR and the 2(-Delta Delta C(T)) method. Methods 25, 402–408. 10.1006/meth.2001.1262 11846609

[B46] Lu J. Meng Z. Cheng B. Liu M. Tao S. Guan S. (2019). Apigenin reduces the excessive accumulation of lipids induced by palmitic acid *via* the AMPK signaling pathway in HepG2 cells. Exp. Ther. Med. 18, 2965–2971. 10.3892/etm.2019.7905 31572539 PMC6755459

[B47] Lu S. Li Y. Qian Z. Zhao T. Feng Z. Weng X. (2023). Role of the inflammasome in insulin resistance and type 2 diabetes mellitus. Front. Immunol. 14, 1052756. 10.3389/fimmu.2023.1052756 36993972 PMC10040598

[B48] Mai B. Han L. Zhong J. Shu J. Cao Z. Fang J. (2022a). Rhoifolin alleviates alcoholic liver disease *in vivo* and *in vitro via* inhibition of the TLR4/NF-κB signaling pathway. Front. Pharmacol. 13, 878898. 35685625 10.3389/fphar.2022.878898 PMC9171502

[B49] Mai B. Han L. Zhong J. Shu J. Cao Z. Fang J. (2022b). Rhoifolin alleviates alcoholic liver disease *in vivo* and *in vitro via* inhibition of the TLR4/NF-κB signaling pathway. Front. Pharmacol. 13, 878898. 10.3389/fphar.2022.878898 35685625 PMC9171502

[B50] Masenga S. K. Kabwe L. S. Chakulya M. Kirabo A. (2023). Mechanisms of oxidative stress in metabolic syndrome. Int. J. Mol. Sci. 24, 7898. 10.3390/ijms24097898 37175603 PMC10178199

[B51] Mcpherron A. C. Guo T. Bond N. D. Gavrilova O. (2013). Increasing muscle mass to improve metabolism. Adipocyte 2, 92–98. 10.4161/adip.22500 23805405 PMC3661116

[B52] Miller G. Schlauch K. Tam R. Cortes D. Torres M. A. Shulaev V. (2009). The plant NADPH oxidase RBOHD mediates rapid systemic signaling in response to diverse stimuli. Sci. Signal 2, ra45. 10.1126/scisignal.2000448 19690331

[B53] Moslehi A. Hamidi-Zad Z. (2018). Role of SREBPs in liver diseases: a mini-review. J. Clin. Transl. Hepatol. 6, 332–338. 10.14218/JCTH.2017.00061 30271747 PMC6160306

[B54] Naomi R. Teoh S. H. Embong H. Balan S. S. Othman F. Bahari H. (2023). The role of oxidative stress and inflammation in obesity and its impact on cognitive Impairments—A narrative review. Anti-oxidants 12, 1071. 10.3390/antiox12051071 PMC10215476 37237937

[B55] Niranjan S. Phillips B. E. Giannoukakis N. (2023). Uncoupling hepatic insulin resistance–hepatic inflammation to improve insulin sensitivity and to prevent impaired metabolism-associated fatty liver disease in type 2 diabetes. Front. Endocrinol. 14, 1193373. 10.3389/fendo.2023.1193373 PMC10313404 37396181

[B56] Noordin L. Nor N. a.M. Bakar N. H. A. Ahmad W. a.N. W. (2021). Metabolic and pancreatic derangement in type 2 diabetic rat. IIUM Med. J. Malays. 20. 10.31436/imjm.v20i4.1917

[B57] Nordmann T. M. Dror E. Schulze F. Traub S. Berishvili E. Barbieux C. (2017). The role of inflammation in β-cell dedifferentiation. Sci. Rep. 7, 6285. 10.1038/s41598-017-06731-w 28740254 PMC5524956

[B58] Novelli E. L. Diniz Y. S. Galhardi C. M. Ebaid G. M. Rodrigues H. G. Mani F. (2007). Anthropometrical parameters and markers of obesity in rats. Lab. Anim. 41, 111–119. 10.1258/002367707779399518 17234057

[B59] Ouyang G. Wang N. Tong J. Sun W. Yang J. Wu G. (2024). Alleviation of taurine on liver injury of type 2 diabetic rats by improving anti-oxidant and anti-inflammatory capacity. Heliyon 10, e28400. 10.1016/j.heliyon.2024.e28400 38560269 PMC10979286

[B60] Panahi Y. Saberi-Karimian M. Valizadeh O. Behnam B. Saadat A. Jamialahmadi T. (2021). Effects of curcuminoids on systemic inflammation and quality of life in patients with colorectal cancer undergoing chemotherapy: a randomized controlled trial. Nat. Prod. Hum. Dis. Pharmacol. Mol. Targets, Ther. Benefits 1328, 1–9. 10.1007/978-3-030-73234-9_1 34981467

[B61] Panchal S. K. Poudyal H. Iyer A. Nazer R. Alam A. Diwan V. (2011). High-carbohydrate, high-fat diet–induced metabolic syndrome and cardiovascular remodeling in rats. J. Cardiovasc. Pharmacol. 57, 51–64. 10.1097/FJC.0b013e3181feb90a 21572266

[B62] Peng S. Hu C. Liu X. Lei L. He G. Xiong C. (2020a). Rhoifolin regulates oxidative stress and proinflammatory cytokine levels in Freund's adjuvant-induced rheumatoid arthritis *via* inhibition of NF-κB. Braz J. Med. Biol. Res. 53, e9489. 32401927 10.1590/1414-431X20209489 PMC7233197

[B63] Peng S. Hu C. Liu X. Lei L. He G. Xiong C. (2020b). Rhoifolin regulates oxidative stress and proinflammatory cytokine levels in Freund’s adjuvant-induced rheumatoid arthritis *via* inhibition of NF-κB. Braz. J. Med. Biol. Res. 53, e9489. 10.1590/1414-431x20209489 32401927 PMC7233197

[B64] Qiu Y. Y. Zhang J. Zeng F. Y. Zhu Y. Z. (2023). Roles of the peroxisome proliferator-activated receptors (PPARs) in the pathogenesis of nonalcoholic fatty liver disease (NAFLD). Pharmacol. Res. 192, 106786. 10.1016/j.phrs.2023.106786 37146924

[B65] Rao Y. K. Lee M.-J. Chen K. Lee Y.-C. Wu W.-S. Tzeng Y.-M. (2011a). Insulin‐mimetic action of rhoifolin and cosmosiin isolated from citrus grandis (L.) osbeck leaves: enhanced adiponectin secretion and insulin receptor phosphorylation in 3T3‐L1 cells. Evidence‐Based Complementary Altern. Med. 2011, 624375. 10.1093/ecam/nep204 PMC3152991 20008903

[B66] Rao Y. K. Lee M. J. Chen K. Lee Y. C. Wu W. S. Tzeng Y. M. (2011b). Insulin-mimetic action of rhoifolin and cosmosiin isolated from citrus grandis (L.) osbeck leaves: enhanced adiponectin secretion and insulin receptor phosphorylation in 3T3-L1 cells. Evid. Based Complement. Altern. Med. 2011, 624375. 10.1093/ecam/nep204 PMC3152991 20008903

[B67] Ravussin E. Smith S. R. Ferrante A. W. Jr. (2021). Physiology of energy expenditure in the weight-reduced state. Obes. (Silver Spring) 29 (Suppl. 1), S31–s38. 10.1002/oby.23095 PMC8988211 33759394

[B68] Redza-Dutordoir M. Averill-Bates D. A. (2016). Activation of apoptosis signalling pathways by reactive oxygen species. Biochim. Biophys. Acta 1863, 2977–2992. 10.1016/j.bbamcr.2016.09.012 27646922

[B69] Rehman H. U. Ullah K. Rasool A. Manzoor R. Yuan Y. Tareen A. M. (2023). Comparative impact of streptozotocin on altering normal glucose homeostasis in diabetic rats compared to normoglycemic rats. Sci. Rep. 13, 7921. 10.1038/s41598-023-29445-8 37193696 PMC10188608

[B70] Rehman K. Akash M. S. H. (2016). Mechanisms of inflammatory responses and development of insulin resistance: how are they interlinked? J. Biomed. Sci. 23, 87–18. 10.1186/s12929-016-0303-y 27912756 PMC5135788

[B71] Saher F. Ijaz M. U. Hamza A. Ain Q. U. Hayat M. F. Afsar T. (2023). Mitigative potential of Rhoifolin against cisplatin prompted testicular toxicity: biochemical, spermatogenic and histological based analysis. Toxicol. Res. (Camb) 12, 814–823. 10.1093/toxres/tfad073 37915485 PMC10615821

[B72] Samuel V. T. Shulman G. I. (2012). Mechanisms for insulin resistance: common threads and missing links. Cell 148, 852–871. 10.1016/j.cell.2012.02.017 22385956 PMC3294420

[B73] Sekiya M. Hiraishi A. Touyama M. Sakamoto K. (2008). Oxidative stress induced lipid accumulation *via* SREBP1c activation in HepG2 cells. Biochem. Biophys. Res. Commun. 375, 602–607. 10.1016/j.bbrc.2008.08.068 18727921

[B74] Shoelson S. E. Lee J. Goldfine A. B. (2006). Inflammation and insulin resistance. J. Clin. investigation 116, 1793–1801. 10.1172/JCI29069 PMC1483173 16823477

[B75] Song D. Yin L. Wang C. Wen X. (2020). Zhenqing recipe attenuates non-alcoholic fatty liver disease by regulating the SIK1/CRTC2 signaling in experimental diabetic rats. BMC Complement. Med. Ther. 20, 27. 10.1186/s12906-019-2811-2 32020874 PMC7076741

[B76] Suh K. S. Oh S. Woo J.-T. Kim S.-W. Kim J.-W. Kim Y. S. (2012). Apigenin attenuates 2-deoxy-D-ribose-induced oxidative cell damage in HIT-T15 pancreatic β-cells. Biol. Pharm. Bull. 35, 121–126. 10.1248/bpb.35.121 22223348

[B77] Sun M. Zhao X. Li X. Wang C. Lin L. Wang K. (2023). Aerobic exercise ameliorates liver injury in Db/Db mice by attenuating oxidative stress, apoptosis and inflammation through the Nrf2 and JAK2/STAT3 signalling pathways. J. Inflamm. Res. 16, 4805–4819. 10.2147/JIR.S426581 37901382 PMC10612520

[B78] Tang W. H. Martin K. A. Hwa J. (2012). Aldose reductase, oxidative stress, and diabetic mellitus. Front. Pharmacol. 3, 87. 10.3389/fphar.2012.00087 22582044 PMC3348620

[B79] Uttarwar L. Gao B. Ingram A. J. Krepinsky J. C. (2012). SREBP-1 activation by glucose mediates TGF-β upregulation in mesangial cells. Am. J. Physiol. Ren. Physiol. 302, F329–F341. 10.1152/ajprenal.00136.2011 22031849

[B80] Vornoli A. Pozzo L. Della Croce C. M. Gervasi P. G. Longo V. (2014). Drug metabolism enzymes in a steatotic model of rat treated with a high fat diet and a low dose of streptozotocin. Food Chem. Toxicol. 70, 54–60. 10.1016/j.fct.2014.04.042 24815820

[B81] Wang Z. Zhu Y. Zhang Y. Zhang J. Ji T. Li W. (2020). Protective effects of AS-IV on diabetic cardiomyopathy by improving myocardial lipid metabolism in rat models of T2DM. Biomed. & Pharmacother. 127, 110081. 10.1016/j.biopha.2020.110081 32244194

[B82] Weber M. Mera P. Casas J. Salvador J. Rodríguez A. Alonso S. (2020). Liver CPT1A gene therapy reduces diet-induced hepatic steatosis in mice and highlights potential lipid biomarkers for human NAFLD. Faseb J. 34, 11816–11837. 10.1096/fj.202000678R 32666604

[B83] World Health Organization (2020). News-room fact-sheets detail obesity and overweight. Available online at: https://www.who.int/newsroom/fact-sheets/detail/obesity-and-overweight .

[B84] Wu L. Guo T. Deng R. Liu L. Yu Y. (2021). Apigenin ameliorates insulin resistance and lipid accumulation by endoplasmic reticulum stress and SREBP-1c/SREBP-2 pathway in palmitate-induced HepG2 cells and high-fat diet–fed mice. J. Pharmacol. Exp. Ther. 377, 146–156. 10.1124/jpet.120.000162 33509902

[B85] Yang Z. Wang L. (2023). Current, emerging, and potential therapies for non-alcoholic steatohepatitis. Front. Pharmacol. 14, 1152042. 10.3389/fphar.2023.1152042 37063264 PMC10097909

[B86] Zengin G. Mostafa N. M. Abdelkhalek Y. M. Eldahshan O. A. (2023a). Anti-oxidant and enzyme inhibitory activities of rhoifolin flavonoid: *in vitro* and *in silico* studies. Chem. & Biodivers. 20, e202300117. 37498319 10.1002/cbdv.202300117

[B87] Zengin G. Mostafa N. M. Abdelkhalek Y. M. Eldahshan O. A. (2023b). Anti-oxidant and enzyme inhibitory activities of rhoifolin flavonoid: *in vitro* and *in silico* studies. Chem. Biodivers. 20, e202300117. 10.1002/cbdv.202300117 37498319

[B88] Zhang J. Li M. N. Yang G. M. Hou X. T. Yang D. Han M. M. (2023). Effects of water-sodium balance and regulation of electrolytes associated with antidiabetic drugs. Eur. Rev. Med. Pharmacol. Sci. 27, 5784–5794. 10.26355/eurrev_202306_32817 37401315

[B89] Zhang L. Ding W.-Y. Wang Z.-H. Tang M.-X. Wang F. Li Y. (2016). Early administration of trimetazidine attenuates diabetic cardiomyopathy in rats by alleviating fibrosis, reducing apoptosis and enhancing autophagy. J. Transl. Med. 14, 109–112. 10.1186/s12967-016-0849-1 27121077 PMC4848862

[B90] Zhang L. Huang Y.-J. Sun J.-P. Zhang T.-Y. Liu T.-L. Ke B. (2021a). Protective effects of calorie restriction on insulin resistance and islet function in STZ-Induced type 2 diabetes rats. Nutr. & metabolism 18, 48. 10.1186/s12986-021-00575-y PMC8097947 33952301

[B91] Zhang L. Huang Y. J. Sun J. P. Zhang T. Y. Liu T. L. Ke B. (2021b). Protective effects of calorie restriction on insulin resistance and islet function in STZ-Induced type 2 diabetes rats. Nutr. Metab. (Lond) 18, 48. 10.1186/s12986-021-00575-y 33952301 PMC8097947

[B92] Zhang Y. Li L. Chai T. Xu H. Du H. Y. Jiang Y. (2024). Mulberry leaf multi-components exert hypoglycemic effects through regulation of the PI-3K/Akt insulin signaling pathway in type 2 diabetic rats. J. Ethnopharmacol. 319, 117307. 10.1016/j.jep.2023.117307 37939911

[B93] Zheng B. Zheng Y. Zhang N. Zhang Y. Zheng B. (2022). Rhoifolin from plumula nelumbinis exhibits anti-cancer effects in pancreatic cancer *via* AKT/JNK signaling pathways. Sci. Rep. 12, 5654. 10.1038/s41598-022-09581-3 35383226 PMC8983741

[B94] Zhou C. Qian W. Li J. Ma J. Chen X. Jiang Z. (2019). High glucose microenvironment accelerates tumor growth *via* SREBP1-autophagy axis in pancreatic cancer. J. Exp. Clin. Cancer Res. 38, 302. 10.1186/s13046-019-1288-7 31296258 PMC6625066

[B95] Zhou X. Zhou J. Ban Q. Zhang M. Ban B. (2024). Effects of metformin on the glucose regulation, lipid levels and gut microbiota in high-fat diet with streptozotocin induced type 2 diabetes mellitus rats. Endocrine 86, 163–172. 10.1007/s12020-024-03843-y 38782861 PMC11445279

